# p53 Deacetylation Alleviates Sepsis-Induced Acute Kidney Injury by Promoting Autophagy

**DOI:** 10.3389/fimmu.2021.685523

**Published:** 2021-07-14

**Authors:** Maomao Sun, Jiaxin Li, Liangfeng Mao, Jie Wu, Zhiya Deng, Man He, Sheng An, Zhenhua Zeng, Qiaobing Huang, Zhongqing Chen

**Affiliations:** ^1^ Department of Critical Care Medicine, Nanfang Hospital, Southern Medical University, Guangzhou, China; ^2^ Guangdong Provincial Key Laboratory of Shock and Microcirculation, School of Basic Medical Sciences, Southern Medical University, Guangzhou, China

**Keywords:** sepsis, acute kidney injury, autophagy, p53, deacetylation

## Abstract

Recent studies have shown that autophagy upregulation can attenuate sepsis-induced acute kidney injury (SAKI). The tumor suppressor p53 has emerged as an autophagy regulator in various forms of acute kidney injury (AKI). Our previous studies showed that p53 acetylation exacerbated hemorrhagic shock-induced AKI and lipopolysaccharide (LPS)-induced endothelial barrier dysfunction. However, the role of p53-regulated autophagy in SAKI has not been examined and requires clarification. In this study, we observed the dynamic changes of autophagy in renal tubular epithelial cells (RTECs) and verified the protective effects of autophagy activation on SAKI. We also examined the changes in the protein expression, intracellular distribution (nuclear and cytoplasmic), and acetylation/deacetylation levels of p53 during SAKI following cecal ligation and puncture (CLP) or LPS treatment in mice and in a LPS-challenged human RTEC cell line (HK-2 cells). After sepsis stimulation, the autophagy levels of RTECs increased temporarily, followed by a sharp decrease. Autophagy inhibition was accompanied by an increased renal tubular injury score. By contrast, autophagy agonists could reduce renal tubular damage following sepsis. Surprisingly, the expression of p53 protein in both the renal cortex and HK-2 cells did not significantly change following sepsis stimulation. However, the translocation of p53 from the nucleus to the cytoplasm increased, and the acetylation of p53 was enhanced. In the mechanistic study, we found that the induction of p53 deacetylation, due to either the resveratrol/quercetin -induced activation of the deacetylase Sirtuin 1 (Sirt1) or the mutation of the acetylated lysine site in p53, promoted RTEC autophagy and alleviated SAKI. In addition, we found that acetylated p53 was easier to bind with Beclin1 and accelerated its ubiquitination-mediated degradation. Our study underscores the importance of deacetylated p53-mediated RTEC autophagy in future SAKI treatments.

## Introduction

Sepsis is defined as organ dysfunction that results from the host’s deleterious response to infection (1). The kidney is one of the most common organs affected by sepsis, resulting in a condition known as sepsis-associated acute kidney injury (also known as sepsis-induced AKI or SAKI), which increased the morbidity and mortality caused by sepsis (2). The accurate estimation of the incidence and trends associated with AKI secondary to sepsis has been challenging. Several cohort studies have described the frequency of AKI among patients with sepsis, and the incidence of SAKI among sepsis patients has been reported between 22% to 53% (3). Even if SAKI patients survive, the possibility of developing chronic kidney disease is greatly increased (2, 3). Our previous studies have shown that damage to renal tubular epithelial cells (RTECs) is an important underlying cause of SAKI (4). However, the exact mechanism of RTEC damage in SAKI is not completely understood.

The tumor suppressor p53 has emerged as an important player in various forms of AKI (5, 6). This transcription factor primarily responds to cellular stress and DNA damage by halting the cell cycle and promoting apoptosis in extreme cases of cell stress including AKI (7). Although the primary role of p53 activation is to safeguard the genome and prevent malignant transformation, its role in AKI appears to be less straightforward and can be detrimental inasmuch as it can trigger cell death in sublethal injured tubular cells (8). Recent evidence indicates that autophagy inhibition is an important cause of various types of AKI (9). The upregulation of p53 could mediate the attenuation of autophagy and aggravate AKI induced by ischemia-reperfusion conditions and cisplatin treatment (6). However, the role of p53 in SAKI has not been reported and the underlying mechanism remains unknown. p53 is subject to a wide range of post-translational modifications (PTM), including phosphorylation, acetylation, methylation, glycosylation, farnesylation, hydroxylation, ADP ribosylation, and PIN1-mediated prolyl isomerization, in addition to modification with ubiquitin and other ubiquitin-like proteins (5). Our previous studies showed that p53 acetylation exacerbated hemorrhagic shock-induced AKI (10) and lipopolysaccharide (LPS)-induced endothelial barrier dysfunction (11).

In this study, we examined the changes in the protein expression, intracellular distribution (nuclear and cytoplasmic), and acetylation/deacetylation state of p53 in cecal ligation and puncture (CLP) or LPS-induced animal model of SAKI and in a LPS-challenged human RTEC cell line (HK-2 cells). Surprisingly, the expression of p53 protein both in renal cortex and HK-2 cell did not significantly change after sepsis stimulation. However, the translocation of p53 from the nucleus to the cytoplasm increased, and the acetylation of p53 was enhanced. In the mechanistic study, we found that the induction of p53 deacetylation, by the activation of deacetylase Sirtuin 1 (Sirt1) through resveratrol/quercetin or the mutation of acetylated lysine site in p53, promoted RTEC autophagy, which alleviated SAKI. In addition, we also found that acetylated p53 was easier to bind with Beclin1 and accelerated its ubiquitination-mediated degradation. Our study underscores the importance of deacetylated p53-mediated RTEC autophagy in future SAKI treatments.

## Material and Methods

### Reagents and Antibodies

Rapamycin (Rapa, S1039), 3-methyladenine (3-MA, S2767), tenovin-6 (S4900), resveratrol (RSV, S1396), chloroquine (CHQ, S6999), quercetin (QCT, S2391), MG132 (S2619) and protease inhibitor cocktail were purchased from Selleck (Houston, Texas, USA). Lipopolysaccharide (LPS, L2630) and pentobarbital (P010) were purchased from Sigma (St. Louis, MO, USA). TUNEL Apoptosis Assay Kit (C1088), deacetylase inhibitor cocktail (P1112), Nuclear and Cytoplasmic Protein Extraction Kit (P0027), and protein A+G agarose beads (P2012) were purchased from Beyotime (Shanghai, China). Immunoprecipitation kit and anti-Beclin1 (11306-1) antibodies were purchased from Proteintech Co. (Chicago, IL, USA). Anti-autophagy-related 5 (Atg5, A0203), anti-p53 (A5761), anti-p62 (A7353), pan-acetylated lysine (A2391), anti-Sirt1 (A0127) and goat anti-mouse lgG (H+L; AS008) antibodies were purchased from Abclonal, China. Anti-microtubule-associated protein 1A/1B-light chain 3 (LC3A/B, 12741S), acetyl-p53 (Lys379; #2570) and Ubiquitin (Ub, P4D1, 3936S) antibodies were purchased from Cell Signaling Technology (Beverly, MA, USA). Anti-glyceraldehyde 3-phosphate dehydrogenase (GAPDH, RM2002), anti-proliferating cell nuclear antigen (PCNA, RM2009), horseradish peroxidase (HRP)-labeled goat anti-mouse secondary antibodies (RM3001), HRP-labeled goat anti-rabbit secondary antibodies (RM3002), and anti-Flag-tag (RM1002) antibodies were purchased from Beijing Ruikang, China. Anti-LC3A/B (SAB1305552) antibody was purchased from Sigma (St. Louis, MO, USA). Anti-p53 (PAb 240) antibody was purchased from Abcam, USA. Antibody against kidney injury molecule-1 (KIM-1, NBP1-76701) was purchased from Novus, USA. TRIzol reagent (#D1105) was purchased from GBCBIO Technologies, China. HiScript^®^ III RT SuperMix (#R323) and SYBR^®^ qPCR Master Mix (#Q331) were purchased from Vazyme, China.

### SAKI Animal Model Study

The experimental animals C57/BL6 mice, weighed between 20–22 g and aged 6–8 weeks, were obtained from the Animal Center of Southern Medical University, Guangzhou, China. The animal experiments were conducted in strict accordance with the recommendations of the Guide for the Care and Use of Laboratory Animals (US National Institutes of Health, Bethesda, MD, USA), and the study protocol was approved by the Committee on Ethics in Animal Experiments of Southern Medical University. Two septic animal models, CLP- and LPS-induced septic mouse models, were used in present study. The chemical reagents with doses of 4 mg/kg Rapa, 20 mg/kg 3-MA, 25 mg/kg tenovin-6, 50 mg/kg CHQ, 30 mg/kg RSV and 25 mg/kg QCT were intraperitoneally injected 2 h before CLP as required, respectively (12–15). All these chemical reagents were first dissolved in DMSO, and then diluted with saline to reach the working concentration.

The CLP procedure was performed as described in previous studies (16). Specifically, the experimental mice were weighed and anesthetized by the intraperitoneal injection of 2% pentobarbital at a dose of 23 mL/kg. A midline laparotomy was performed using mini-midsection, and the cecum was ligated just below the ileocecal valve by using 4–0 silk ligatures to maintain intestinal continuity. The cecum was perforated at 2 locations 1 cm apart by using a 21-gauge needle and gently compressed until feces were extruded. The bowel was then returned to the abdomen, and the incision was closed. Control mice (sham group) underwent the same surgical procedures without ligating and puncturing the cecum. At the end of the operation, all mice received fluid resuscitation with normal saline (50 mL/kg) by subcutaneous injection. After the CLP model was created, the mice were executed for the observation of renal pathological changes. The autophagy in RETCs, the expression level of p53 protein in kidney and serum creatinine (sCr) levels were also detected. Some other animals (20 in each group) were remained for survival analyses.

LPS-induced septic mouse model was created with 15 mg/kg LPS *via* intraperitoneal injection as described in previous study (17). The mice were then executed for the observation of the autophagy in RETCs and the expression level of p53 protein in kidney.

### Cell Culture, Transfection, and Adenovirus Transduction

The experimental RTEC HK-2 cells were purchased from Kunming Cell Bank, China (numbered KCB200815YJ). HK-2 cells were cultured in Dulbecco’s modified Eagle’s medium (DMEM) supplemented with 10% (v/v) heat-inactivated fetal bovine serum (FBS) at 37°C in a humidified atmosphere containing 5% CO_2_. A recent mycoplasma contamination test on HK-2 cells was negative.

HK-2 cells were stimulated with 10 µg/ml LPS to construct a cellular sepsis model. According to the requirements of different experiments, cells were transfected with control small interfering RNA (siRNA), Sirt1 siRNA, empty plasmid vectors, HA-Sirt1 plasmid and His-Ub plasmid vectors for 6 h by using Opti-MEM^®^ I reduced serum media and Lipofectamine 2000. The siRNA, HA-SIRT1 and His-Ub plasmid were synthesized by GenePharma (Shanghai, China). The siRNA-targeted sequences were as follows: control siRNA, sense 5’-UUCUCCGAACGUGUCACGUTT-3’ and antisense 5’-ACGUGACACGUUCGGAGAATT-3’; SIRT1 siRNA, sense 5’- GCUGUACGAGGAGAUAUUUTT-3’ and antisense 5’- AAAUAUCUCCUCGUACAGCTT-3’. After the cells were transfected for 36 h, follow-up experiments were performed. The adenoviral vector expressing Flag- and GFP-tagged wild-type and lysine-mutated p53 (Ad-p53 and Ad-p53K382R, respectively) were packaged by GeneChem Co. Ltd (Shanghai, China). The adenoviral vector expressing Flag- and GFP-tagged wild-type Beclin1 (Ad-Beclin1) was also packaged by GeneChem Co. Ltd. Cells were transduced with 10^9^ plaque-forming units (PFU)/mL of adenoviruses for 48 h, followed by corresponding experiments.

### Histology and Immunohistochemistry

The histology of mouse kidneys was observed by hematoxylin-eosin (HE) staining and periodic acid-Schiff (PAS) staining. Briefly, fresh renal cortical tissues were sliced into sections, fixed with 10% formalin for approximately 24 h, and then stained and observed under an optical microscope (Zeiss, LSM780, Thuringia Germany). Two professional pathologists, who were blinded to the experimental protocol and the experimental group of this subject, scored the damage to the renal cortex. Five sections were selected for each group, and 10 random fields of view were selected from each section. The histological assessment of renal damage included the observed loss of the tubule brush border, renal tubule hemorrhage, tubular casts, inflammatory infiltration, and obvious necrosis in the cortex (18, 19). The scoring principle was as follows: 0, no damage; 1, ≤10% damage; 2, 11%–25% damage; 3, 26%–45% damage; 4, 46%–75% damage, and 5, ≥76% damage. Finally, statistical analysis and graphing were performed, according to the scoring results.

For immunohistochemical staining, renal tissues were obtained, fixed with 4% formaldehyde solution overnight, dehydrated using an alcohol gradient, cleared using xylene, and embedded in paraffin. Subsequently, the paraffin-embedded tissue blocks were sliced into 4–6 µm sections, which were then mounted onto glass slides. The slides were baked in an oven at 55°C for 2 h, followed by deparaffinization and rehydration, prior to antigen retrieval using sodium citrate. KIM-1 has been used as an useful biomarker for renal proximal tubule damage to facilitate the early diagnosis of renal tubule dysfunction (20). In this study, kidney sections were probed with antibodies against KIM-1 at a dilution of 1:100 at room temperature for 1 h. Then the sections were incubated for 15 min at room temperature with biotinylated secondary antibody. A streptavidin-peroxidase enzyme conjugate was added to each section for 10 min, and the peroxidase activity of KIM-1 was visualized. The density of KIM-1 staining was quantified by Image software (Media Cybernetics, Inc., Rockville, MD, USA)

### TUNEL Assay

Terminal deoxynucleotidyl transferase dUTP mediated nick-end labeling (TUNEL) assay was performed to evaluate apoptosis in renal tissues, as described in our previously published literature (16). Briefly, the renal tissues (4 µm) were fixed, paraffin-embedded, and labeled with a TUNEL reaction mixture containing terminal deoxynucleotidyl transferase and nucleotides including tetramethylrhodamine-labeled dUTP. After the reactions were terminated, the slides were examined by fluorescence microscopy. Blue dots, generated by 4’, 6-diamidino-2-phenylindole (DAPI) staining, identified the nucleus, whereas green dots were generated by the TUNEL assay, indicating the presence of degraded DNA. The overlap between the blue dots and green dots represented apoptotic cells. A total of 30 fields of view from 5 samples were used to evaluate the number of TUNEL-positive cells in the kidney. Data are expressed as the number of apoptotic cells in each 200× magnification field of view.

### Renal Function Assessment

Blood samples were collected at 12 h after CLP and then centrifuged at 3,000 rpm for 15 min at room temperature to separate the serum. The levels of sCr were analyzed using an automatic biochemical analyzer (Chemray 240, Shenzhen, China).

### Ultrastructure Observation With Transmission Electron Microscopy

Kidney tissues were cryosectioned in 2% formaldehyde and 2.5% glutaraldehyde in 0.1 M sodium cacodylate buffer for 1 h and then washed in phosphate-buffered saline (PBS) for 2 days. The samples were then fixed in 1% osmic acid, dehydrated with ethanol and acetone gradients, embedded in epoxy resin, and cut into ultrathin sections. The tissue sections were stained with uranyl acetate and lead citrate and then observed under an H-7500 transmission electron microscope (Hitachi, Tokyo, Japan). The number of autophagosomes and autophagolysosomes was counted in 30 randomly selected fields in each group at 8000× magnification, and representative fields at 40,000× magnification were selected for display.

### Western Blotting Analysis

The kidney tissues and cells were homogenized by ultrasound and lysed in radioimmunoprecipitation assay (RIPA) lysis buffer containing 1× protease inhibitor cocktail. To detect the acetylation level of the target protein, 1× deacetylase inhibitor cocktail was added to the RIPA lysis buffer. The samples were then centrifuged at 12,000 rpm for 15 min to obtain the supernatants. The proteins in the supernatants were separated using sodium dodecyl sulfate (SDS)-polyacrylamide gel and transferred to polyvinylidene difluoride (PVDF) membranes. The PVDF membrane was blocked in 5% bovine serum albumin (BSA) for 1 h to remove the specific binding and then incubated with primary antibodies overnight at 4°C and secondary antibodies at room temperature for 1 h, and finally, the target protein was visualized with enhanced chemiluminescence reagents. The primary antibodies used were anti-Atg5, anti-LC3A/B, anti-p53, pan-acetylated lysine, acetyl-p53 (Lys379), anti-Sirt1, anti-p62, anti-Beclin1, Anti-GAPDH, anti-PCNA, anti-Flag-tag antibodies. The secondary antibodies used were HRP-labeled goat anti-mouse and anti-rabbit antibodies. The band intensities were quantified using ImageJ software. The protein expression levels were standardized relative to the level of GAPDH or PCNA or p53.

### Autophagic Flux Detection by mRFP-GFP-LC3 Adenovirus Transfection

mRFP-GFP-LC3 adenovirus (Hanbio Co. Ltd., Shanghai, China) was transfected to HK-2 cells in each confocal cuvette at a titer of 10^10^ PFU/mL for 48 h, then the cells were harvested and fixed in 4% paraformaldehyde. The expression of both monomeric red fluorescent protein (mRFP) and green fluorescent protein (GFP) in the mRFP-GFP-LC3 tandem fluorescent proteins were used to track LC3 under a confocal laser microscope. Generally, when autophagosomes form in the cells, LC3 will converge into dots, and the LC3 dots will be tagged with both mRFP, resulting in red fluorescence and GFP, resulting in green fluorescence, simultaneously appearing yellow due to superimposition. However, GFP is sensitive to acidity. When autophagosomes fuse with acidic lysosomes, forming autophagolysosomes, the GFP protein becomes quenched, and the LC3 spots only appear red. Therefore, autophagy in HK-2 cells can be recognized by counting the yellow and red dots. In this study, the numbers of spots of each color were obtained from at least three independent experiments in each group.

### Immunoprecipitation and Co-Immunoprecipitation Assays

The enriched protein was incubated with 2 µg anti-p53 or anti-Beclin1 antibody overnight at 4°C. Then 40 µl of protein A+G agarose beads were added and spined at 4°C for 3 h. After centrifugation, the supernatant was discarded, and 1× loading buffer was added for western blotting analysis to determine protein expression. The primary antibodies used were pan-acetylated lysine, anti-Beclin1 and Ubiquitin antibodies. The protein expression levels were standardized relative to the level of p53 or IgG-H.

### Nucleus and Cytoplasm Protein Isolation

A Nuclear and Cytoplasmic Protein Extraction Kit was used to isolate the nuclear and cytoplasmic p53 proteins from HK-2 cells. According to the manufacturer’s recommendations, adherent cells were scraped, and the cell pellet was obtained by centrifugation. Then, 200 µl cytoplasmic protein extraction reagent A containing PMSF was added for every 20 µl of cell pellet volume. After the cell pellet was completely dispersed by vortexing at the highest speed for 5 s, the sample was placed in an ice bath for 10–15 min. Then, 10 µl of cytoplasmic protein extraction reagent B was added to the cell pellet. The mixture was vortexed at the highest speed for 5 s again and placed in an ice bath for another 1 min, followed by centrifugation at 14,000 × *g* at 4°C for 5 min to obtain the supernatant, which was retained as the cytoplasmic protein fraction. Then, 50 µl of nuclear protein extraction reagent containing PMSF was added to the remaining cell pellet. The mixture was subjected to an ice bath and vortexed at high speed for 30 s every 1–2 min for a total of 30 min. Finally, the cell pellet was centrifuged at 14,000 × *g* at 4°C for 10 min to obtain the nuclear protein. The nuclear and cytoplasmic proteins can be used immediately or stored at −70°C.

### Immunofluorescence Assays

HK-2 cells were inoculated onto Petri dishes. After discarding the original medium, the cells were rinsed three times with PBS, fixed with 4% paraformaldehyde for 15 min, punched with 0.5% Triton-100 for 15 min, blocked with 5% BSA for 1 h, and then incubated overnight at 4°C with primary antibodies for p53 or LC3 at a dilution of 1:100. The next day, fluorescent secondary antibodies were added and incubated at room temperature for 1 h, and then the nucleus was stained with DAPI after 3 washes with PBS.

### Survival Study

Sepsis was induced by CLP, as described above. After awakening from anesthesia, the mice were returned to their cages, and food and water were provided. For survival analysis, 20 mice were included in each group (sham group, vehicle + CLP group and RSV + CLP group), and mice were monitored daily over a 5-day period, and the survival rate was recorded. Survival after surgery was assessed every 6 h during the first 48 h and then every 8 h for 3 days. Apnea for >1 min was considered to indicate death. Mice that survived for > 5 d were sacrificed by cervical dislocation.

### Real-Time Quantitative Reverse Transcription Polymerase Chain Reaction (RT-qPCR) Assays

Total RNA was isolated from cells with TRIzol reagent. The RNA concentration was determined by using a BioDrop spectrophotometer (Biochrom Ltd, Cambridge, UK). Complementary DNA was reverse transcribed with HiScript^®^ III RT SuperMix according to the manufacturer’s protocol. RT- PCR was performed using SYBR^®^ qPCR Master Mix with specific primers (**Table 1**) in a 7500 Real-Time PCR System (Applied Biosystems, Foster City, CA). β-actin was used as the internal control. The 2−ΔΔCt method was employed to evaluate mRNA expression.

**Table 1:** Sequences of primers for RT-qPCR.

Gene name	Forward primer	Reverse primer
*ATG2A*	5’-CCTCTGTGAGACCAAGGATGAG-3’	5’-CCAGACAGAAGTAGCCAAGTCC-3’
*ATG2B*	5’-CTTCAGATGGAGTTGGAGGAGAC-3’	5’-AGTGGCTCCTTTCAGTCCTACG-3’
*ATG4B*	5’-ATGGGAGTTGGCGAAGGCAAGT-3’	5’-AGCTCCACGTATCGAAGACAGC-3’
*TSC2*	5’-GCCACTACTGTGCCTTTGAGTC-3’	5’-CCCTCAGAGAATCGCCAGTACT-3’
*UVRAG*	5’-TCTACACCGACAACTCCATCCG-3’	5’-TCTGGCATTTTGGAGAGGAAGTG-3’
*DRAM1*	5’-ATCTTCAAGCCATCCTGTGTG-3’	5’-GAGGTTTGATCCGCATAATCTG-3’
*BAX*	5’-TCAGGATGCGTCCACCAAGAAG-3’	5’-TGTGTCCACGGCGGCAATCATC-3’
*PIG3*	5’-CACCAGTTTGCTGAGGTCTAGG-3’	5’-CCTGGATTTCGGTCACTGGGTA-3’
*NOXA1*	5’-CTCGATGCAGAGACAGAGGTCG-3’	5’-AGGAGCCTGTTTGCCAACTTGC-3’
*β-Actin*	5’-AAATCGTGCGTGACATCAAAGA-3’	5’-GCCATCTCCTGCTCGAAGTC -3’

### Ubiquitination Assay

For detection of ubiquitinated proteins, cells were transfected with Ad-Beclin1 along with Ad-p53 or Ad-p53K382R. After 12 h, 4 μg of His-Ub expression plasmid was transfected into the cells. After 36 h of transfection, 5 μM MG132, the proteasome inhibitor, was added along with LPS and the cells were further incubated for 12 h. Cells were then harvested for immunoprecipitation.

### Statistical Analysis

Survival analysis was performed using Kaplan-Meier plots and compared using the log-rank test. Other results were expressed as the mean ± standard deviation and analyzed by one-way analysis of variance (ANOVA) with SPSS 20.0 (IBM, Armonk, NY, USA). Variance in groups was evaluated using the homogeneity of variance test (the Levene test). When the Levene test indicated homogeneity of variance (*P > 0.1*), the least-significant difference multiple comparison test was used. When equal variance was not assumed (the Levene test; *P < 0.1*), the Welch method was applied, followed by Dunnett’s T3 *post hoc* comparisons. All tests were performed using SPSS software (Chicago, IL). P values *< 0.05* were considered significant.

## Results

### Autophagy Inhibition Precedes Apoptosis in RTECs Following Sepsis

**Figure 1:**
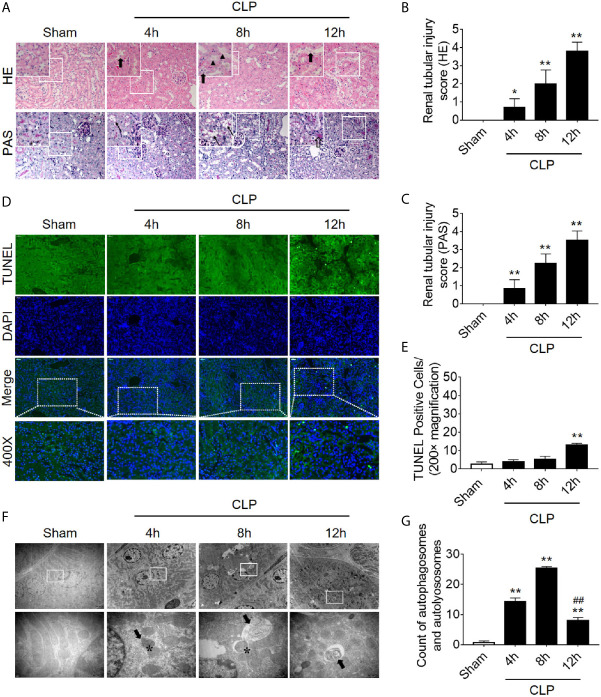
Renal pathology score, apoptosis, and autophagy following CLP-induced sepsis. **(A)** Hematoxylin-eosin (HE) staining (upper panel: 200×; inset: 400×) and periodic acid-Schiff (PAS) staining (lower panel: 200×; inset: 400×) of the renal cortex following CLP-induced sepsis. Black thick arrows: Nuclei of RTECs shed to lumen; Long black arrows: tubular dilation; Black triangles: renal tubular casts; White thick arrows: severe tubular damage. **(B, C)** The tubular damage score was evaluated based on pathological observations from HE and PAS staining. *p < 0.05, ^**^ *p* < 0.01 *vs.* sham group; n = 5. **(D)** Cell apoptosis of the renal cortex, as assessed by TUNEL-positive cell staining following CLP-induced sepsis. Upper panel: 200×; lower panel: 400×. **(E)** Semi-quantitative analysis of TUNEL-positive cells. ^**^ *p* < 0.01 *vs.* sham group; n = 5. **(F)** Observation of autophagy in the renal cortex under transmission electron microscope following CLP-induced sepsis. Black thick arrows: autophagosomes or autolysosomes; Asterisk: mitochondrial autophagy; upper panel: magnified ×7000; lower panel: magnified ×40000. **(G)** Semi-quantitatively analysis of autophagy. The numbers of autophagosomes and autolysosomes in renal epithelial cells were calculated in 20 randomly selected fields. ^**^ *p* < 0.01 *vs.* sham group; ^##^ *p* < 0.01 *vs.* 8 h group; n = 20. CLP, cecal ligation and puncture; RTEC, renal tubular epithelial cell; TUNEL, terminal deoxynucleotidyl transferase dUTP nick-end labeling.

We first dynamically observed damage in kidney tissues in the CLP-induced AKI model. Both HE staining and PAS staining showed that some renal tubules expanded and swelled within 4 h after CLP, and some RTECs fell off into the lumen. These pathological changes were gradually aggravated over the next 12 h post-CLP, and the renal tubular injury scores were correspondingly elevated (**Figures 1A–C**). Interestingly, the apoptosis level, as assessed by TUNEL staining, only increased significantly after 12 h of CLP (**Figures 1D, E**). Transmission electron microscopy observation and the quantitative analysis of autophagosomes and autolysosomes revealed that autophagy in RTECs increased progressively and then sharply decreased within 8 h after CLP. The decline in autophagy levels preceded the occurrence of apoptosis (**Figures 1F, G**). Subsequently, we also measured the dynamic changes of autophagy in LPS-induced septic mouse model. We found that the expression of autophagy-related protein LC3II increased gradually and peaked at 8 h, returned to baseline by 24 h. In contrast, p62 (SQSTM1, a mediator of cargo selection and an autophagic substrate) expression reached the lowest levels at 8 h with LPS treatment (**Supplementary Figures 1A–C**), which was consistent with the autophagosomes analysis in CLP-induced septic mouse model. Similar changes of LC3II and p62 have been reported in CLP model in our previous study (15).

**Figure 2:**
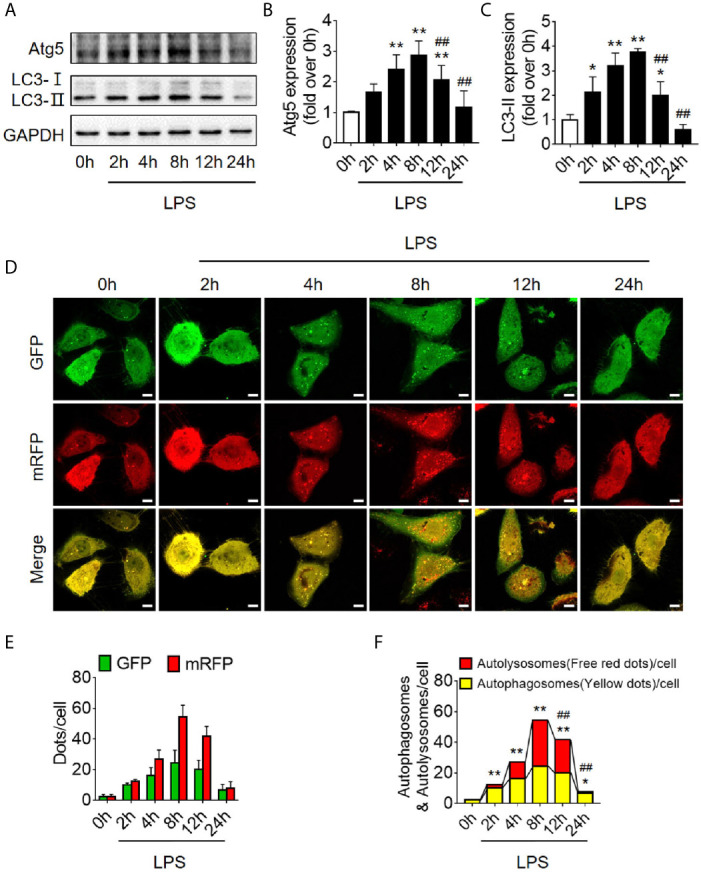
Autophagy determination in LPS-induced HK-2 cells. **(A)** Representative western blot showing the Atg 5 and LC3II protein expression levels in HK-2 cells following LPS stimulation at various time-points. GAPDH was used as an internal reference. **(B, C)** densitometric analyses of Atg5 and LC3II protein expression. n = 4-5. **(D)** The autophagic flux in HK-2 cells was determined by cellular immunoassay following LPS stimulation at various time-points. The autophagy double-labeled adenovirus mRFP-GFP-LC3 was used to detect autophagic flux (magnification ×630 and scale bar = 10 µm). **(E)** The mean number of GFP and mRFP dots per cell. **(F)** The mean numbers of autophagosomes and autolysosomes per cell. ^*^ *p* < 0.05, ^**^ *p* < 0.01 *vs.* the 0 h group, ^##^ *p* < 0.01 *vs.* the 8 h group. The results are representative of at least three independent experiments. LPS, lipopolysaccharide; Atg5, autophagy-related protein 5; LC3II, Microtubule-associated protein 1A/1B-light chain 3; GAPDH, glyceraldehyde 3-phosphate dehydrogenase; mRFP, monomeric red fluorescence protein; GFP, Green fluorescent protein.

Based on the findings of animal experiments, we further detected autophagy in LPS-challenged human RTECS (HK-2 cells). The expression of autophagy-related protein Atg5 and LC3II gradually increased and reached a peak at 8 h and then sharply decreased and dropped at 24 h (**Figures 2A–C**). Considering that the hindrance of autophagic flux would cause the failure of autophagosomes to fuse with lysosomes to form autophagolysosomes, which would result in the increased expression of autophagy-related proteins; therefore, we detected autophagic flux using laser confocal microscopy. We transfected HK-2 cells with the autophagy double-labeled adenovirus mRFP-GFP-LC3 to detect autophagic flux. According to the manufacturer’s recommendations, free red dots [red dots (mRFP) minus the overlap of red and green dots (GFP)] represented autolysosomes, and the yellow dots with overlapping red dots and green dots represented autophagosomes. As expected, the number of both autophagosomes and autophagolysosomes showed a trend of increasing first and then decreasing (**Figures 2D–F**), indicating that the autophagy flux has not been blocked, which is the same as the observed trend of autophagy-related protein expression.

### Autophagy Activation Alleviates Renal Injury

**Figure 3:**
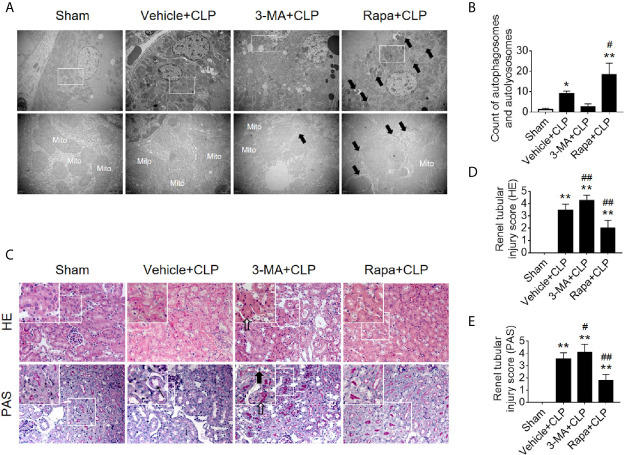
Effects of autophagy regulation on renal pathology score and autophagy in CLP-induced sepsis. **(A)** Observation of autophagic structures in the renal cortex under a transmission electron microscope following autophagy regulation in septic mice. Black thick arrows: autophagosomes or autolysosomes; Mito, mitochondria; upper panel: magnified ×7,000; lower panel: magnified ×40,000. **(B)** Semi-quantitatively analysis of autophagy. The numbers of autophagosomes and autolysosomes in renal epithelial cells were calculated in 20 randomly selected fields. ^**^ *p* < 0.05, ^**^ *p* < 0.01 *vs.* sham group; ^#^ *p* < 0.05 *vs.* the vehicle + CLP group; n = 20. **(C)** Autophagy regulation on Hematoxylin-Eosin (HE) staining (upper panel: 200×; inset: 400×) and periodic acid-Schiff (PAS) staining (lower panel: 200×; inset: 400×) of the renal cortex following CLP-induced sepsis. Black thick arrows: Nuclei of RTECs shed to lumen; Long black arrows: tubular dilation; White thick arrows: severe tubular damage. **(D, E)** The tubular damage score was evaluated based on pathological observations from HE and PAS staining. ^*^ *p* < 0.01 *vs.* sham group; ^#^ *p* < 0.05, ^##^ *p* < 0.01 *vs.* the vehicle + CLP group; n = 5. CLP, cecal ligation and puncture; RTEC, renal tubular epithelial cell.

Next, we verified the protective effects of autophagy activation on kidney after sepsis. The autophagy agonist Rapa and the autophagy inhibitor 3-MA were used for this experiment. Using transmission electron microscopy, we found that Rapa pretreatment significantly promoted the level of autophagy in RTECs at 12 h after CLP (**Figures 3A, B**) and alleviated renal tubular injury (**Figures 3C–E**). Correspondingly, 3-MA inhibited the formation of autophagosomes in RTECs, which further aggravated renal tubular injury after CLP (**Figure 3**). A more specific autophagy inhibitor CHQ was applied to further confirm the effect of autophagy inhibition. The result showed that CHQ blocked autophagy with significant increase of p62 expression (**Supplementary Figures 2A–C**), accompanied by aggravated renal tubular injury (**Supplementary Figures 2D, E**).

### Translocation of p53 From Nucleus to Cytoplasm Increases Following Sepsis

**Figure 4:**
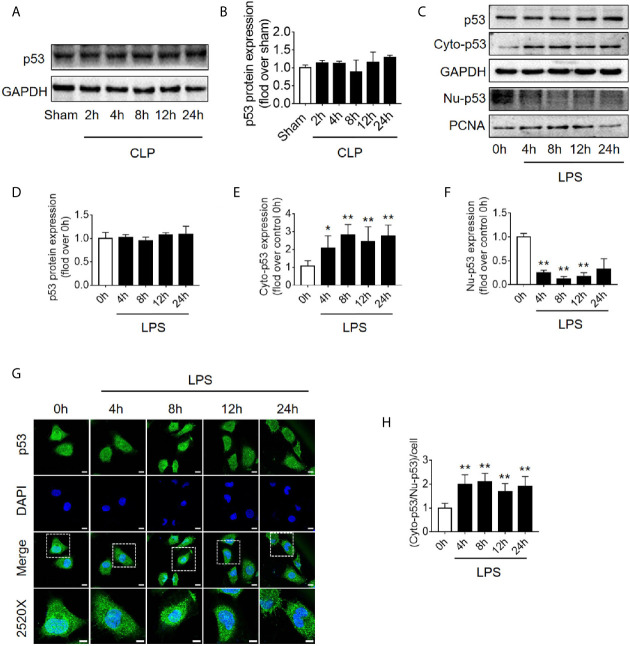
p53 protein expression and intracellular distribution. **(A)** Representative western blot showing p53 protein levels in the renal cortex following CLP-induced sepsis. GAPDH was used as an internal reference. **(B)** Densitometric analyses of p53 protein expression. n = 3. **(C)** Representative western blot showing p53 protein levels in HK-2 cells following LPS treatment. GAPDH was used as an internal control of p53 total protein and cytoplasmic protein levels, and PCNA was used as an internal control for nuclear proteins. **(D–F)** Densitometric analyses of p53 total protein level, cytoplasmic protein level (cyto-p53), and nuclear protein level (Nu-p53). n = 3-5. ^*^ *p* < 0.05, ^**^ *p* < 0.01 *vs.* the 0 h group. **(G)** Representative cellular immunofluorescence observation of p53 in the nucleus and cytoplasm after LPS stimulation. p53 was labeled with green fluorescence using FITC, and the nucleus was labeled with blue fluorescence by DAPI. The magnification of the upper panel is 630×, and the magnification of the bottom panel is 2520×. Scale bar, 10 µm. **(H)** The ratio of the fluorescence intensity of cytoplasm p53 to nucleus p53 in each cell. ^**^ *p* < 0.01 *vs.* the 0 h group. Results represent at least three independent experiments. CLP, cecal ligation and puncture; GAPDH, glyceraldehyde 3-phosphate dehydrogenase; LPS, lipopolysaccharide; PCNA, proliferating cell nuclear antigen; FITC, fluorescein isothiocyanate; DAPI, 4’,6-diamadino-2-phenylindole.

Because the level of apoptosis was increased while autophagy started to be inhibited (**Figure 1**), it is speculated that autophagy inhibition might be induced by signaling changes in apoptosis pathway. p53, an important molecule in crosstalk of autophagy and apoptosis, was emerged. First, we found that the expression level of p53 protein in renal cortical tissue was not elevated (**Figures 4A, B**). The pro-autophagic role of p53 on renal tubular cells has been shown to depend on the intracellular distribution of p53 (6). We then measured the protein abundance of p53 in the nucleus and cytoplasm of HK-2 cells stimulated by LPS. We found that although the total p53 protein level was not changed significantly, the nuclear p53 protein level was progressively reduced, and correspondingly, the cytoplasmic level of p53 progressively increased, suggesting an increase in p53 nuclear to cytoplasmic translocation after 4–12 h of LPS stimulation (**Figures 4C–F**). These phenomena were further confirmed in cellular immunofluorescence experiments (**Figures 4G, H**). Since p53 works as a transcription factor while it locates in nucleus, the expression of p53 target genes, selectively those autophagy and apoptosis pathway-related genes were detected. The results revealed that, as p53 translocated out of nucleus, autophagy-related ATG2A mRNA was decreased continuously. ATG2B and ATG4B mRNA level were transiently increased at LPS stimulation but then decreased, while DRAM1, UVRAG and TSC2 did not change (**Supplementary Figures 3A–F**). The mRNA expression of pro-apoptotic BAX, PIG3, and NOXA1 were decreased 8 h after LPS stimulation (**Supplementary Figures 3G–I**). These results were mostly coincidence with the timing of p53 translocation out of nucleus.

### Increased p53 Acetylation Inhibits Autophagy in RTECs

**Figure 5:**
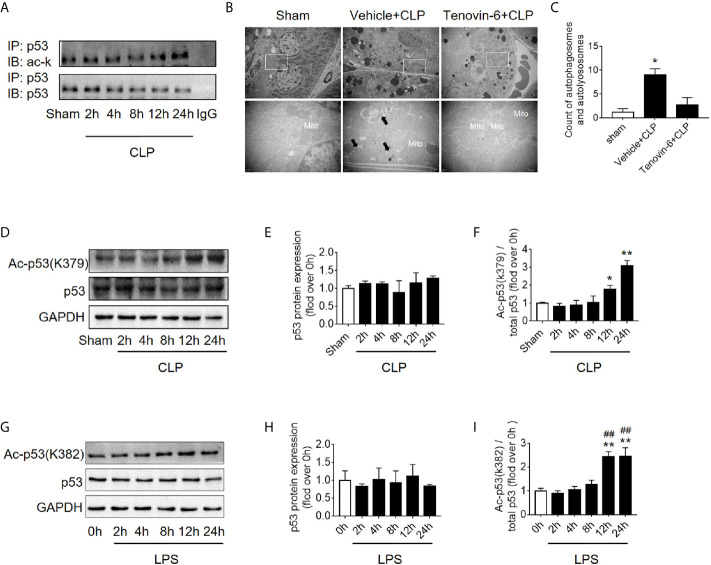
p53 acetylation following CLP/LPS-induced sepsis. **(A)** Protein expression and acetylation levels of p53 based on p53 immunoprecipitation (IP) from the kidney cortex of CLP-induced septic mice. IB, immunoblotting; ac, acetylation. **(B)** p53 activation of autophagy in the renal cortex of CLP-induced septic mice. Tenovin-6 was used to upregulate p53 acetylation. Black thick arrows: autophagosomes or autolysosomes; Mito: mitochondria; upper panel: magnified ×7,000; lower panel: magnified ×40,000. **(C)** Semi-quantitative analysis of autophagy. The numbers of autophagosomes and autolysosomes in renal epithelial cells were calculated in 20 randomly selected fields. ^*^ *p* < 0.05 *vs.* sham group. **(D)** Levels of total protein expression and acetylated p53 (ac-p53) at lysine site K379 in the kidney cortex of CLP-induced septic mice. **(E, F)** Densitometric analyses of the levels of p53 protein expression and acetylation (ac-p53) at lysine site of K379 in the kidney cortex of CLP-induced septic mice. ^*^ *p* < 0.05, ^**^ *p* < 0.01 *vs.* sham group; n = 3-4. **(G)** Levels of total protein expression and acetylated p53 (ac-p53) at lysine site K382 in LPS-induced HK-2 cells. **(H, I)** Densitometric analyses of the levels of p53 protein expression and acetylation at lysine site K382 in LPS-induced HK-2 cells. ^**^ *p* < 0.01 *vs.* the 0 h group; ^##^ *p* < 0.01 *vs.* the 8 h group; n = 4. CLP, cecal ligation and puncture; LPS, lipopolysaccharide.

It is known that p53 acetylation modification is critical in its activity, we then explored the level of p53 acetylation during SAKI and examined the effects of promoting p53 acetylation on autophagy. As expected, the acetylation of p53 in renal cortical tissue was considerably increased (**Figure 5A**) at 12 h following CLP. According to previous reports (5, 6) and our published data (10, 11), p53 acetylation may increase p53 activity. Tenovin-6 acts as an enhancer of endogenous acetylation of p53 by inhibiting the protein-deacetylating activities of Sirt1 and Sirt2 (21, 22). It is used in this study to induce p53 acetylation in CLP septic model. As expected, tenovin-6 treatment significantly increased p53 acetylation at 12 h after CLP (**Supplementary Figures 1G–H**), reduced the number of autophagosomes in RTECs and exacerbated kidney damage (**Figures 5B, C**). Since the acetylation of p53 (acetyl-p53) aggravated the autophagy in RTECs after sepsis were confirmed, the specific acetylation site deserves further clarification. According to a previous report (20), the acetylation site of p53 is K379 in mice and K382 in humans. Our study showed that the acetyl-p53 (K379) was significantly increased in the renal cortical tissues at 12 h after CLP/LPS compared with the level in the control group (**Figures 5D–F** and **Supplementary Figures 1D–F**). Consistent with the animal model, the acetyl-p53 (K382) level in HK-2 cells was also considerably increased 12 h following the LPS challenge (**Figures 5G–I**). In our study, the translocation of p53 from nucleus to cytoplasm increased after 4–12 h of LPS stimulation, so we also measured the acetylation levels of cytoplasmic p53 and nuclear p53 respectively. There was an increasing trend in acetylation of cytoplasmic p53 8 h after LPS exposure (**Supplementary Figures 4A–C**).

### Mutation of Lysine (K) of p53 to Arginine (R) With Loss of PTM Site Promotes Autophagy

**Figure 6:**
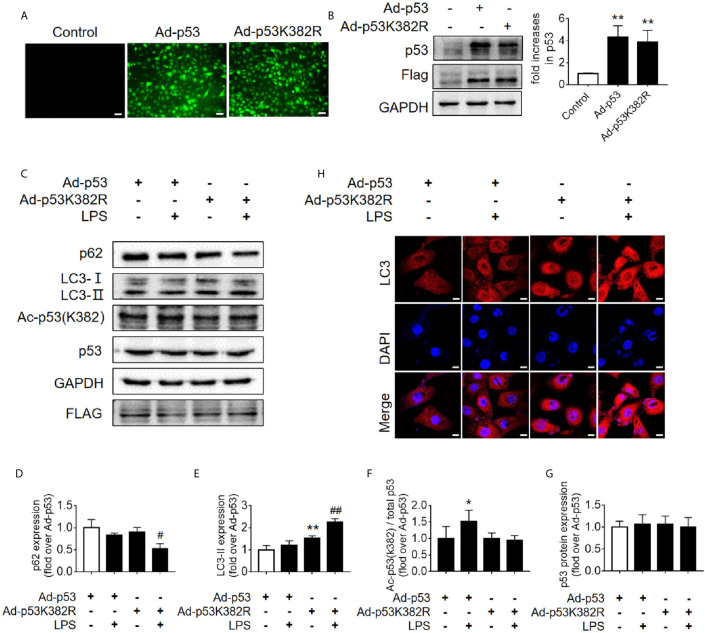
Mutation of p53 (K382 lysine site) mediated-p53 deacetylation promotes autophagy. **(A)** Confirmation of the effect of p53 virus transfection. HK-2 cells were transfected with either p53 adenovirus (Ad-p53) or p53K382R adenovirus (Ad-p53K382R). Deacetylation of p53 was induced by the mutation of lysine (K) at 382 to arginine (R). **(B)** Representative western blot and densitometric analyses of p53 protein expression. n = 3. ^**^ *p* < 0.01 *vs.* Control group. **(C)** Representative western blot of p62, LC3-I, LC3-II, acetylated p53 (Ac-p53), and p53. GAPDH was used as an internal reference. **(D–G)** Densitometric analyses of p62, LC3-I, LC3-II, acetylated p53 (Ac-p53), and p53. n = 3-4. ^*^ *p* < 0.05, ^**^ *p* < 0.01 *vs.* the Ad-p53 group; ^#^ *p* < 0.05, ^##^ *p* < 0.01 *vs.* the Ad-p53 + LPS group. **(H)** Effects of the p53 point mutation on autophagy protein LC3 under cellular immunofluorescence (magnification ×630 and scale bar = 10 µm). LC3 is labeled with red fluorescence of GRF, and the nucleus is labeled with the blue fluorescence of DAPI. The brightness of red represents the abundance of LC3 protein. LC3II, Microtubule-associated protein 1A/1B-light chain 3; GAPDH, glyceraldehyde 3-phosphate dehydrogenase; LPS, lipopolysaccharide; DAPI, 4’, 6-diamadino-2-phenylindole.

Since the K382 lysine is the main acetylation site of p53 in human cells, we mutated this site to explore the effects of p53 deacetylation on autophagy promotion. We used p53 overexpression virus (Ad-p53) and p53 K382R mutant virus (Ad-p53K382R) with both GFP and Flag tags. The transfection efficiency of the virus was verified by examining the expression levels of GFP and p53 (**Figures 6A, B**). Compared with the Ad-p53+LPS group, no elevated acetyl-p53 was observed in the Ad-p53K382R+LPS group after LPS stimulation due to the site mutation, whereas the level of p62 protein significantly decreased (**Figures 6C–G**), and LC3II protein increased, as assessed by western blotting (**Figures 6C–G**) and immunofluorescence analysis (**Figure 6H**). These results collectively suggested that the deacetylation of p53 *via* a point mutation at the lysine K382 could promote autophagy. These data confirmed the influence of p53 acetylation/deacetylation on autophagy regulation. Therefore, the identification of a drug capable of regulating p53 deacetylation could be useful for promoting autophagy in SAKI.

### Activation of Deacetylase Sirt1 Promotes Autophagy and Reduces SAKI

**Figure 7:**
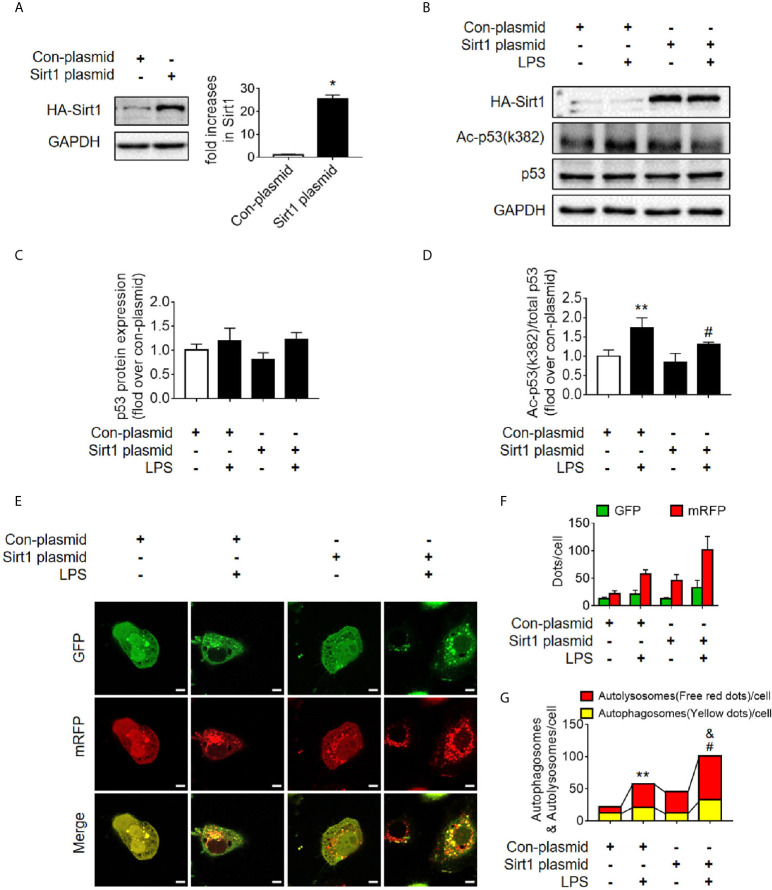
Determination of the effects of Sirt1 plasmid transfection. **(A)** Confirmation of plasmid-induced Sirt1 overexpression using western blot. Con-plasmid, control plasmid. **(B)** Representative western blot of HA-Sirt1, acetylated-p53 (Ac-p53), and p53 in LPS-treated HK-2 cells. **(C, D)** Densitometric analyses of p53 protein expression and acetylated-p53 (Ac-p53). n = 3-4. **(E)** Effects of Sirt1 overexpression on autophagic flux based on the cellular immunoassay following LPS stimulation at different groups (magnification ×630 and scale bar = 10 µm). **(F)** Effects of Sirt1 overexpression on GFP and mRFP counts per cell. **(G)** Effects of Sirt1 overexpression on autophagosomes and autolysosomes per cell. *p < 0.05, ^**^ *p* < 0.01 *vs.* con-plasmid group; ^#^ *p* < 0.05 *vs.* con-plasmid + LPS group; ^&^ *p* < 0.05 *vs.* the Sirt1 plasmid group. The results are representative of at least three independent experiments. LPS, lipopolysaccharide; GFP, green fluorescent protein; mRFP, monomeric red fluorescent protein.

**Figure 8:**
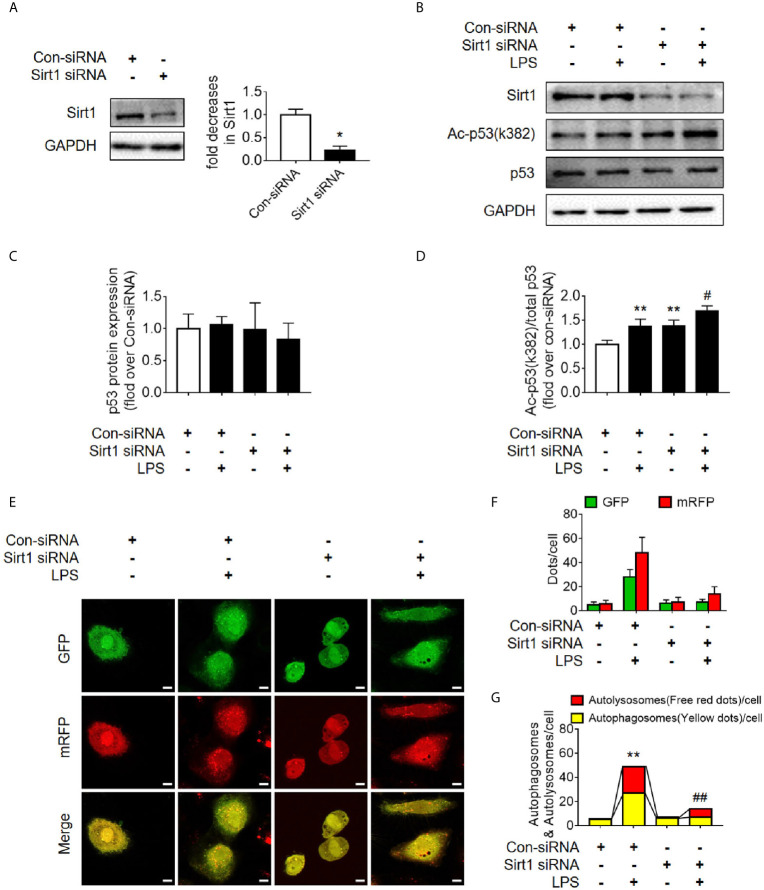
Determination of the effects of Sirt1 small interfering RNA (siRNA) transfection. **(A)** Confirmation of siRNA-mediated Sirt1 knockdown using western blot. Con-siRNA, control siRNA. **(B)** Representative Western blot of Sirt1, acetylated-p53 (Ac-p53), and p53 in LPS-treated HK-2 cells. **(C, D)** Densitometric analyses of p53 protein expression and acetylated-p53 (Ac-p53). n = 3. **(E)** Effects of Sirt1 knockdown on autophagic flux assessed by cellular immunoassay following LPS stimulation in different groups (magnification ×630 and scale bar = 10 µm). **(F)** Effect of Sirt1 knockdown on the numbers of GFP and mRFP counts per cell. **(G)** Effect of Sirt1 knockdown on autophagosomes and autolysosomes per cell. *p < 0.05, ^**^ *p* < 0.01 *vs.* con-siRNA group; ^#^ *p* < 0.05, ^##^ *p* < 0.01 *vs.* con-siRNA + LPS group. Results represent at least three independent experiments. LPS, lipopolysaccharide; GFP, green fluorescent protein; mRFP, monomeric red fluorescent protein.

Sirt1 is a NAD^+^-dependent deacetylase that is known to directly deacetylate p53 (K382) to regulate the function of p53 (23). To investigate the effects of Sirt1-mediated deacetylation of p53 on autophagy, HA-tagged Sirt1 overexpression plasmid (**Figure 7A**) and Sirt1 siRNA (**Figure 8A**) were applied in RTECs. As expected, Sirt1 overexpression did not decrease the expression of total p53 and the translocation of p53 from nucleus to cytoplasm, but significantly reduced the level of acetyl-p53 (K382) in HK-2 cells challenged with LPS for 12 h (**Figures 7B–D** and **Supplementary Figures 4D–F**). By contrast, siRNA-mediated Sirt1 knockout aggravated p53 acetylation but did not affect p53 protein expression (**Figures 8B–D**). Moreover, Sirt1 overexpression elevated the numbers of both autophagosomes and autolysosomes (**Figures 7E–G**), whereas Sirt1 knockout attenuated the formation of autophagosomes and autolysosomes (**Figures 8E–G**). These results indicated that Sirt1-induced deacetylation of p53 was able to enhance RTEC autophagy.

**Figure 9:**
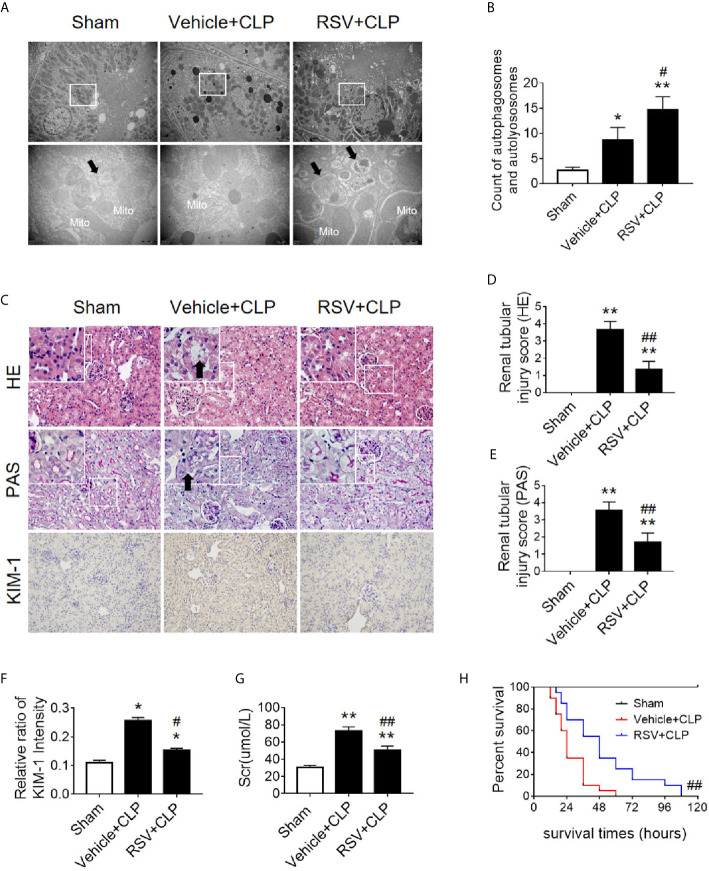
Activation of the deacetylase Sirt1 by RSV promotes autophagy and reduces SAKI. **(A)** Effect of Sirt1 activation by RSV on autophagy in the renal cortex of CLP-induced septic mice. Black thick arrows: autophagosomes or autolysosomes; Mito, mitochondria. Upper panel: magnified ×7,000; lower panel: magnified ×40,000. **(B)** Semi-quantitative analysis of autophagy. The number of autophagosomes and autolysosomes in renal epithelial cells was calculated in 20 randomly selected fields. **(C)** Effects of Sirt1 activation by RSV on kidney pathology, as evidenced by HE staining, PAS staining, and KIM-1 immunohistochemistry. Upper Panel: Hematoxylin-Eosin (HE) staining (200×; inset: 400×); Middle panel: periodic acid-Schiff (PAS) staining (200×; inset: 400×). Black thick arrows: Nucleus of RTECs shed to lumen; Lower panel: KIM-1 immunohistochemistry (200×). **(D, E)** The tubular damage score was evaluated based on pathological observations from HE and PAS staining. These scores are based on the data obtained from the observation of 5 specimens in each group with 10 randomly selected fields of view from a 200× microscope for each specimen. **(F)** Relative ratio of kidney injury molecule-1 (KIM-1). The data were obtained from at least three independent experiments. **(G)** Effect of Sirt1 activation by RSV on the level of sCr. **(H)** Effects of Sirt1 activation by RSV on the survival times in CLP-induced septic mice. The survival rates were estimated by the Kaplan-Meier method and compared by the log-rank test. n = 20. ^*^ *p* < 0.05, ^**^ *p* < 0.01 *vs.* sham group; ^#^ *p* < 0.05, ^##^ *p* < 0.01 *vs.* the vehicle + CLP group. CLP, cecal ligation and puncture; RTEC, renal tubule epithelial cell.

Two Sirt1 chemical agonists, RSV and QCT, were then applied to clarify the effects of Sirt1 activation on acetylation of p53 and RTEC autophagy (24). First, we verified that both RSV and QCT could reduce the acetylation level of p53 at lysine 379 (**Supplementary Figures 5A–F**). Then we found that RSV administration promoted the autophagy of RTECs, as determined by an observed increase in autophagosomes (**Figures 9A, B**). Consistently, QCT administration increased the protein expression of LC3II, but decreased the protein expression of p62 (**Supplementary Figures 5G–I**). Moreover, RSV attenuated pathological renal damage (**Figure 9C**), resulted in a reduced renal tubular damage score (**Figures 9D, E**), and partially reduced the levels of KIM-1 (**Figures 9C, F**) and sCr (**Figure 9G**). QCT also attenuated pathological kidney damage (**Supplementary Figures 2D–F**). In addition, RSV administration prolonged the survival times of CLP mice (**Figure 9H**).

### p53 Interacted With Beclin1 and Acetylated p53 Promoted Ubiquitination of Beclin1

**Figure 10:**
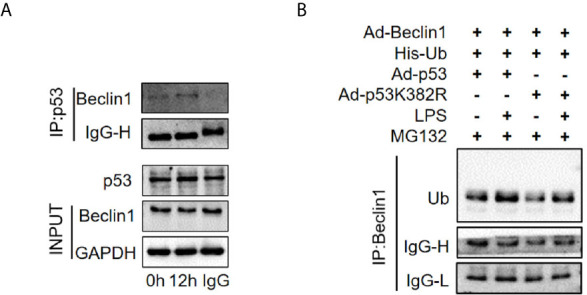
The interaction of p53 and Beclin1 in HK-2 cells. **(A)** Association of Beclin1 with p53 in the HK-2 cells. The interaction between p53 and Beclin1 was determined by Co- immunoprecipitation assays. **(B)** Determination of the ubiquitination of Beclin1. Ad-Beclin1 and His-tagged ubiquitin protein and Ad-p53 or Ad-p53K382R were individually transfected into HK-2 cells with or without LPS stimulation. LPS, lipopolysaccharide.

Beclin1 is a well-known key regulator of autophagy. Our previous studies have shown that the protein level of Beclin1 is reduced in the late stage of SAKI (15), so we supposed that acetylated p53 might regulate autophagy through Beclin1. The interaction of p53 and Beclin1 was first confirmed in our study (**Figure 10A**) and then we found that transfection of p53 K382R mutant virus significantly reduced the ubiquitination of Beclin1 in HK-2 cells (**Figure 10B**). These results suggested that acetylated p53 suppressed the autophagy by promoting the ubiquitination-mediated degradation of Beclin1.

## Discussion

**Figure 11:**
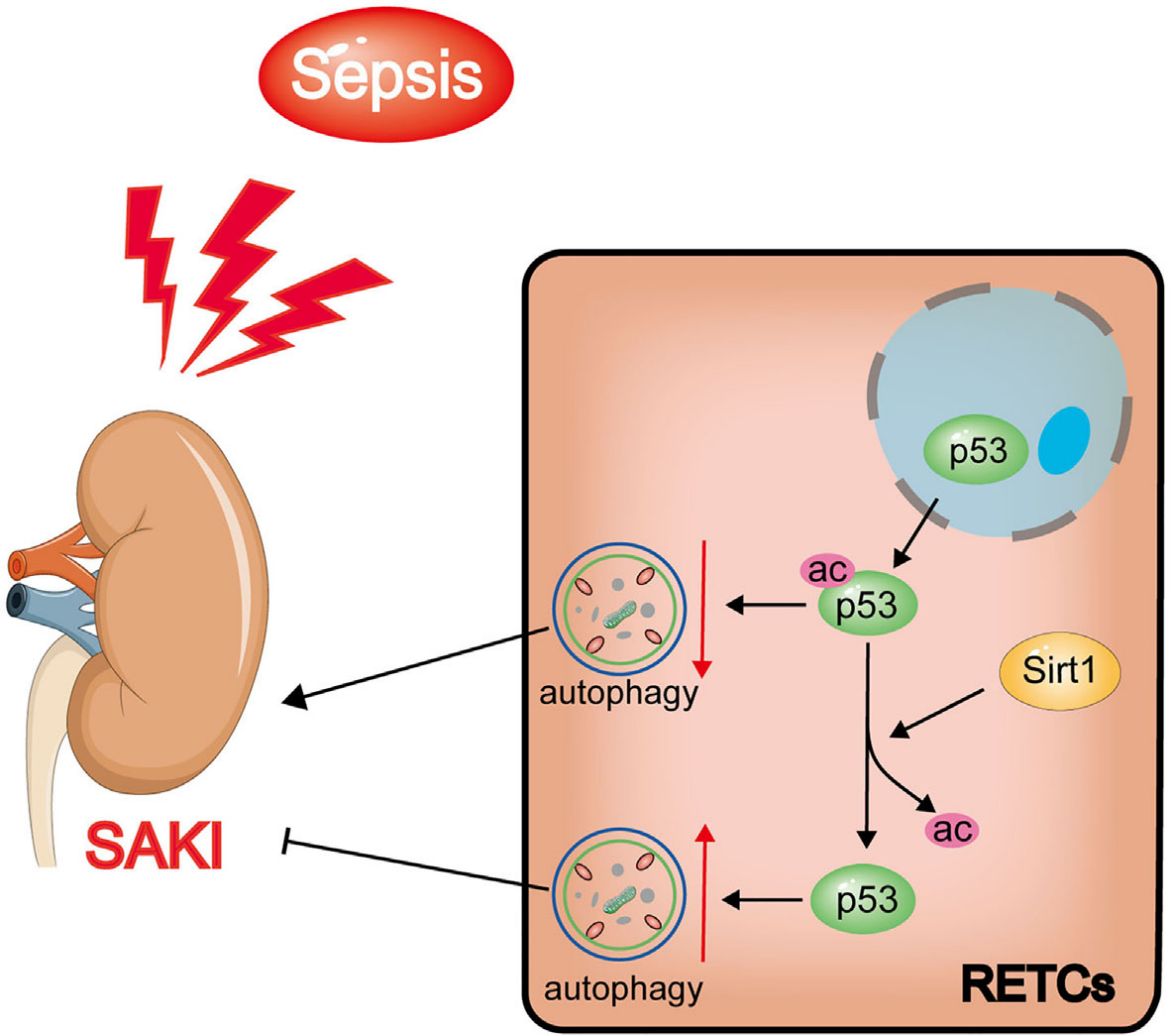
Schematic diagram showing the effects of Sirt1-induced p53 deacetylation on autophagy activation in septic-associated acute kidney injury (SAKI). The p53 total protein expression does not change significantly, but the nuclear to cytoplasmic translocation and acetylation increased in renal tubular epithelial cells (RTECs) following sepsis. The elevated acetylation of p53 promotes autophagy inhibition and aggravates SAKI. The deacetylation of p53 induced by Sirt1 can attenuate this process to a certain extent.

In this study, we found that increased levels of p53 acetylation suppressed RTEC autophagy after sepsis. The activation of autophagy, which was induced by p53 following deacetylation by Sirt1, was able to reduce SAKI (**Figure 11**). Our study provides a new perspective for elucidating the underlying mechanisms of SAKI, indicating a possible avenue for intervention and identifying a future drug target.

Autophagy, another type of programmed cell death different from apoptosis, occurs in all eukaryotic cells and contributes to the renewal and reuse of cellular components and energy homeostasis (25). The process of autophagy is complex, and its hallmark is the formation of autophagosomes and autophagolysosomes. During this process, autophagy-related proteins such as Beclin1 and LC3 both increase, while the autophagy cargo receptor protein p62 decreases (26). A previous study demonstrated that the LIR domain enables p62 to combine with LC3II, and the UBA domain enables p62 to bind to ubiquitin; consequently, ubiquitinated proteins may be degraded by autophagy. In SAKI, excessive accumulation of p62 is able to promote apoptosis, to enhance the release of the toxic substance LDH, and to inhibit the proliferation of RTECs (21). Different from the final result of cell death caused by apoptosis, activated autophagy plays a renal protective role by preventing apoptosis, preserving the mitochondrial functions, and maintaining the balance between the productions of pro- and anti-inflammatory cytokines (22). Our previous research has confirmed the above phenomena and verified that autophagy activation could reduce SAKI (15). The role of p53 in SAKI is rarely reported, although previous studies have explored the damaging role of p53 in bilateral renal ischemia-reperfusion induced AKI and cisplatin nephrotoxic AKI (27, 28). Other literature has suggested that acetylated p53 may cause damage to myocardial cells (29), liver cells (30), and neurons (31) under septic conditions by promoting apoptosis. Garrison et al. (32) suggested that p53 mediates apoptosis to optimize the neutrophil lifespan and ensure the proper clearance of bacteria, presenting a counter-balance between the innate immune response to infection and survival following DNA damage. Our research confirmed that the acetylation of p53 in RTECs aggravated SAKI. Histological examination found that RTEC is less likely to undergo apoptosis and necrosis during the development of SAKI (33, 34). In consistent with this finding, we found that p53 might not act through the apoptotic pathway but rather acts through the inhibition of autophagy during the pathogenesis of SAKI. Our results revealed particularly significant inhibition of autophagy 12 h after CLP, indicating that the influence of p53 on the development of SAKI might rely on the regulation of autophagy rather than the promotion of apoptosis, which represents the largest innovation of this research to the understanding of the mechanisms that drive SAKI.

Interestingly, a large body of previous literature (30, 35, 36) and our own previous studies (4, 10, 11) have shown that an increase in p53 activity may be related to the modification of its acetylation swfi 2tatus. Acetylated p53 can be deacetylated by various deacetylases, such as histone deacetylase-1 (HDAC1), HDAC6 and Sirt1. In research mainly related to tumors, it has been reported that HDAC1 and HDAC6 could deacetylate p53, leading to the repression of p53-dependent transcriptional activation, cell growth arrest, as well as apoptosis (37–39). The role of p53 in sepsis depends on its acetylation/deacetylation modifications, which is primarily regulated by the newly identified deacetylation modification enzyme Sirt1. Sirt1 is a nicotinamide adenine dinucleotide (NAD+) dependent protein deacetylase, which was originally found to regulate apoptosis and DNA repair, which affects longevity (40). Sirt1 deacetylates both histones and other non-histone proteins (10). Our previous studies have demonstrated that Sirt1 can improve SAKI by deacetylating Beclin1, which mediates autophagy activation (15). The first non-histone target that was identified for Sirt1 activity was p53. Our previous study revealed that Sirt1 protein expression also decreased gradually following sepsis (41), which should be the cause of p53 acetylation in this study. Present study further emphasized the role of p53 deacetylation in autophagy activation and provided an experimental basis for the more precise exploration of p53 deacetylation modifications (the non-Sirt1-mediated broad-spectrum deacetylation) as potential future treatments for SAKI.

Previous studies have shown that the regulatory effects of p53 on autophagy are closely related to the intracellular distribution of p53. Nuclear p53 transactivates a large set of target genes involved in the autophagic process, including AMP-activated protein kinase (AMPK) (6, 42). In a rat AKI model, induced by ischemia-reperfusion injury, the nuclear p53 localization in renal tubular cells was demonstrated to promote autophagy (43). However, cytosolic p53 may have an anti-autophagic function (44). In many studies (45–47), cytosolic p53 inhibited mitophagy (a common form of autophagy) by disturbing the mitochondrial translocation of Parkin. In this study, we found that although the total protein expression level of p53 did not change significantly during the early stages of SAKI, the nuclear to cytoplasmic translocation of p53 increased prior to the observed inhibition of autophagy. We also found that, coincident with the translocation of p53 out of nucleus, the mRNA levels of p53 target genes ATG2A, ATG4B, BAX, PIG3A and NOXA1 decreased, while the mRNA levels of ATG2B, DRAM1, UVRAG and TSC2 did not change significantly. In addition, the interaction of p53 and Beclin1 has also been confirmed in this study. In HK-2 cells stimulated by LPS for 12 h, wild-type p53 virus significantly ubiquitinated Beclin1, while p53K382R mutant virus reduced the ubiquitination of Beclin1. Therefore we speculated that acetylated p53 promoted the proteasomal degradation of Beclin1, leading to the suppression of autophagy in HK-2 cells. This result also provides another pathway or mechanism for the effect of Sirt1, showing that the Sirt1-induced alteration of p53 acetylation status might also in turn affect the activity of Beclin1 and then autophagy. In fact, previous studies have proved that p53 and beclin-1 interact through the BH3 domain of beclin-1, and the cytoplasm is the main site of interaction (48). However, the mechanism underlying the translocation of p53 from nuclear to cytoplasmic remains unclear, and this will also be an important direction for future research.

In this study, we applied RSV and QCT to activate Sirt1 to deacetylate p53. The administration of RSV and QCT both promoted the autophagy of RTECs and attenuated pathological renal damage in septic model. It is noticed that because of the persistent presence of CLP-induced infection in mice (feces in the abdominal cavity were not cleared or the intestinal contents may continue to drain into the abdominal cavity), the mice eventually die. Therefore, the administration of RSV only prolonged the survival time of CLP mice, which is consistent with other studies (49, 50).

Our research has some limitations. First, the regulatory mechanism underlying the p53 nuclear to cytoplasmic translocation process has not yet been studied. Second, the index in this study did not involve apoptosis, especially the p53-related apoptosis pathways. Instead, we focused on the mechanism through which p53 acetylation/deacetylation regulates autophagy. Third, a more precise study of the effect of p53 acetylation on autophagy by transfection of adenovirus will help increase the depth and credibility of our research and the observation time could be extended (such as more than 24 h or even longer). Moreover, we also need to consider the use of p53 virus or recombinant protein in septic mice model.

In conclusion, our study found that p53 acetylation-mediated autophagy inhibition underlies the pathogenesis of SAKI. We demonstrated that Sirt1 upregulation could reduce SAKI by deacetylating p53 to activate autophagy, and Sirt1 activators, such as resveratrol or quercetin, might have potential to be developed into a SAKI treatment in the future.

## Data Availability Statement

The original contributions presented in the study are included in the article/**Supplementary Material**. Further inquiries can be directed to the corresponding authors.

## Ethics Statement

The animal study was reviewed and approved by Committee on Ethics in Animal Experiments of Southern Medical University.

## Author Contributions

ZC, QH, and ZZ prepared the concept and designed the research. MS, JL, LM, JW, ZD, MH, and SA performed the experiments. ZC, ZZ, and MS analyzed data. MS and JL prepared the figures. MS, LM, ZZ, and QH interpreted the results of experiments. MS and JL drafted the paper. ZC, QH, and ZZ edited and revised the paper. MS and JL contributed equally to this paper. All authors contributed to the article and approved the submitted version.

## Funding

This work was supported by the National Natural Science Foundation of China [grant numbers 81701955, 81871604 and 81870210], the Natural Science Foundation of Guangdong Province, China [grant numbers 2020A151501361 and 2017A030313590], and the Guangdong Basic and Applied Basic Research Foundation [grant number 2019A1515012022].

## Conflict of Interest

The authors declare that the research was conducted in the absence of any commercial or financial relationships that could be construed as a potential conflict of interest.

## References

[1] Singer M Deutschman CS Seymour CW Shankar-Hari M Annane D Bauer M. The Third International Consensus Definitions for Sepsis and Septic Shock (Sepsis-3). Jama (2016) 315:801–10. 10.1001/jama.2016.0287 PMC4968574 26903338

[2] Poston JT Koyner JL. Sepsis Associated Acute Kidney Injury. BMJ (Clinical Res ed) (2019) 364:k4891. 10.1136/bmj.k4891 PMC6890472 30626586

[3] Bellomo R Kellum JA Ronco C Wald R Martensson J Maiden M. Acute Kidney Injury in Sepsis. Intensive Care Med (2017) 43:816–28. 10.1007/s00134-017-4755-7 28364303

[4] Xu S Gao Y Zhang Q Wei S Chen Z Dai X. SIRT1/3 Activation by Resveratrol Attenuates Acute Kidney Injury in a Septic Rat Model. Oxid Med And Cell Longevity (2016) 2016:7296092. 10.1155/2016/7296092 PMC5149703 28003866

[5] Kruiswijk F Labuschagne CF Vousden KH. P53 in Survival, Death and Metabolic Health: A Lifeguard With a Licence to Kill. Nat Rev Mol Cell Biol (2015) 16:393–405. 10.1038/nrm4007 26122615

[6] Tang C Ma Z Zhu J Liu Z Liu Y Liu Y. P53 in Kidney Injury and Repair: Mechanism and Therapeutic Potentials. Pharmacol Ther (2019) 195:5–12. 10.1016/j.pharmthera.2018.10.013 30347214

[7] Molitoris BA Dagher PC Sandoval RM Campos SB Ashush H Fridman E. siRNA Targeted to P53 Attenuates Ischemic and Cisplatin-Induced Acute Kidney Injury. J Am Soc Nephrol JASN (2009) 20:1754–64. 10.1681/ASN.2008111204 PMC2723992 19470675

[8] Sutton TA Hato T Mai E Yoshimoto M Kuehl S Anderson M. P53 Is Renoprotective After Ischemic Kidney Injury by Reducing Inflammation. J Am Soc Nephrol JASN (2013) 24:113–24. 10.1681/ASN.2012050469 PMC3537213 23222126

[9] Zhao W Zhang L Chen R Lu H Sui M Zhu Y. SIRT3 Protects Against Acute Kidney Injury via AMPK/mTOR-Regulated Autophagy. Front Physiol (2018) 9:1526. 10.3389/fphys.2018.01526 30487750 PMC6246697

[10] Zeng Z Chen Z Xu S Zhang Q Wang X Gao Y. Polydatin Protecting Kidneys Against Hemorrhagic Shock-Induced Mitochondrial Dysfunction via SIRT1 Activation and P53 Deacetylation. Oxid Med Cell Longevity (2016) 2016:1737185. 10.1155/2016/1737185 PMC4783550 27057271

[11] Zhang W Zhang Y Guo X Zeng Z Wu J Liu Y. Sirt1 Protects Endothelial Cells Against LPS-Induced Barrier Dysfunction. Oxid Med Cell Longevity (2017) 2017:4082102. 10.1155/2017/4082102 PMC5676476 29209448

[12] Wang Y Quan F Cao Q Lin Y Yue C Bi R. Quercetin Alleviates Acute Kidney Injury by Inhibiting Ferroptosis. J Advanced Res (2021) 28:231–43. 10.1016/j.jare.2020.07.007 PMC7753233 33364059

[13] Yang M Cao L Xie M Yu Y Kang R Yang L. Chloroquine Inhibits HMGB1 Inflammatory Signaling and Protects Mice From Lethal Sepsis. Biochem Pharmacol (2013) 86:410–8. 10.1016/j.bcp.2013.05.013 PMC3713089 23707973

[14] Singh D Chander V Chopra K. The Effect of Quercetin, a Bioflavonoid on Ischemia/Reperfusion Induced Renal Injury in Rats. Arch Med Res (2004) 35:484–94. 10.1016/j.arcmed.2004.10.004 15631872

[15] Deng Z Sun M Wu J Fang H Cai S An S. SIRT1 Attenuates Sepsis-Induced Acute Kidney Injury via Beclin1 Deacetylation-Mediated Autophagy Activation. Cell Death Dis (2021) 12:217. 10.1038/s41419-021-03508-y 33637691 PMC7910451

[16] Wei S Gao Y Dai X Fu W Cai S Fang H. SIRT1-Mediated HMGB1 Deacetylation Suppresses Sepsis-Associated Acute Kidney Injury. Am J Physiol Renal Physiol (2019) 316:F20–f31. 10.1152/ajprenal.00119.2018 30379096

[17] Wu J Deng Z Sun M Zhang W Yang Y Zeng Z. Polydatin Protects Against Lipopolysaccharide-Induced Endothelial Barrier Disruption via SIRT3 Activation. Lab Investigation; A J Tech Methods Pathol (2020) 100:643–56. 10.1038/s41374-019-0332-8 31641228

[18] Zhao X Qiu X Zhang Y Zhang S Gu X Guo H. Three-Dimensional Aggregates Enhance the Therapeutic Effects of Adipose Mesenchymal Stem Cells for Ischemia-Reperfusion Induced Kidney Injury in Rats. Stem Cells Int (2016) 2016:9062638. 10.1155/2016/9062638 26649053 PMC4663369

[19] Jiang Y Zeng Y Huang X Qin Y Luo W Xiang S. Nur77 Attenuates Endothelin-1 Expression via Downregulation of NF-κb and P38 MAPK in A549 Cells and in an ARDS Rat Model. Am J Of Physiol Lung Cell Mol Physiol (2016) 311:L1023–l1035. 10.1152/ajplung.00043.2016 27765761 PMC5206403

[20] Krummel KA Lee CJ Toledo and G.M. Wahl F. The C-Terminal Lysines Fine-Tune P53 Stress Responses in a Mouse Model But Are Not Required for Stability Control or Transactivation. Proc Natl Acad Sci USA (2005) 102:10188–93. 10.1073/pnas.0503068102 PMC1177381 16006521

[21] Li T Zhao J Miao S Xu Y Xiao X Liu Y. Dynamic Expression and Roles of Sequestome−1/P62 in LPS−induced Acute Kidney Injury in Mice. Mol Med Rep (2018) 17:7618–26. 10.3892/mmr.2018.8809 PMC5983950 29620262

[22] Dai XG Xu W Li T Lu JY Yang Y Li Q. Involvement of Phosphatase and Tensin Homolog-Induced Putative Kinase 1-Parkin-Mediated Mitophagy in Septic Acute Kidney Injury. Chin Med J (2019) 132:2340–7. 10.1097/CM9.0000000000000448 PMC6819035 31567378

[23] Vaziri H Dessain SK Ng Eaton E Imai SI Frye RA Pandita TK. Hsir2(SIRT1) Functions as an NAD-Dependent P53 Deacetylase. Cell (2001) 107:149–59. 10.1016/S0092-8674(01)00527-X 11672523

[24] Wang D He X Wang D Peng P Xu X Gao B. Quercetin Suppresses Apoptosis and Attenuates Intervertebral Disc Degeneration via the SIRT1-Autophagy Pathway. Front In Cell Dev Biol (2020) 8:613006. 10.3389/fcell.2020.613006 33363176 PMC7758489

[25] Cuervo AM Bergamini E Brunk UT Dröge W Ffrench M Terman A. Autophagy and Aging: The Importance of Maintaining "Clean" Cells. Autophagy (2005) 1:131–40. 10.4161/auto.1.3.2017 16874025

[26] Kaushal GP Shah SV. Autophagy in Acute Kidney Injury. Kidney Int (2016) 89:779–91. 10.1016/j.kint.2015.11.021 PMC4801755 26924060

[27] Cao JY Wang B Tang TT Wen Y Li ZL Feng ST. Exosomal miR-125b-5p Deriving From Mesenchymal Stem Cells Promotes Tubular Repair by Suppression of P53 in Ischemic Acute Kidney Injury. Theranostics (2021) 11:5248–66. 10.7150/thno.54550 PMC8039965 33859745

[28] Yang A Liu F Guan B Luo Z Lin J Fang W. P53 Induces miR-199a-3p to Suppress Mechanistic Target of Rapamycin Activation in Cisplatin-Induced Acute Kidney Injury. J Cell Biochem (2019) 120:17625–34. 10.1002/jcb.29030 31148231

[29] Han D Li X Li S Su T Fan L Fan WS. Reduced Silent Information Regulator 1 Signaling Exacerbates Sepsis-Induced Myocardial Injury and Mitigates the Protective Effect of a Liver X Receptor Agonist. Free Radical Biol Med (2017) 113:291–303. 10.1016/j.freeradbiomed.2017.10.005 28993270

[30] Lee Y Jeong GS Kim KM Lee W Bae JS. Cudratricusxanthone A Attenuates Sepsis-Induced Liver Injury via SIRT1 Signaling. J Cell Physiol (2018) 233:5441–6. 10.1002/jcp.26390 29226969

[31] Zhao L An R Yang Y Yang X Liu H Yue L. Melatonin Alleviates Brain Injury in Mice Subjected to Cecal Ligation and Puncture via Attenuating Inflammation, Apoptosis, and Oxidative Stress: The Role of SIRT1 Signaling. J Pineal Res (2015) 59:230–9. 10.1111/jpi.12254 26094939

[32] Garrison SP Thornton JA Häcker H Webby R Rehg JE Parganas E. The P53-Target Gene Puma Drives Neutrophil-Mediated Protection Against Lethal Bacterial Sepsis. PloS Pathog (2010) 6:e1001240. 10.1371/journal.ppat.1001240 21203486 PMC3009602

[33] Gomez H Ince C De Backer D Pickkers P Payen D Hotchkiss J. A Unified Theory of Sepsis-Induced Acute Kidney Injury: Inflammation, Microcirculatory Dysfunction, Bioenergetics, and the Tubular Cell Adaptation to Injury. Shock (Augusta Ga) (2014) 41:3–11. 10.1097/SHK.0000000000000052 PMC3918942 24346647

[34] Hotchkiss RS Karl IE. The Pathophysiology and Treatment of Sepsis. New Engl J Med (2003) 348:138–50. 10.1056/NEJMra021333 12519925

[35] Ma H Wang X Ha T Gao M Liu L Wang R. MicroRNA-125b Prevents Cardiac Dysfunction in Polymicrobial Sepsis by Targeting TRAF6-Mediated Nuclear Factor κb Activation and P53-Mediated Apoptotic Signaling. J Infect Dis (2016) 214:1773–83. 10.1093/infdis/jiw449 PMC5144735 27683819

[36] Zhang H Xu CF Ren C Wu TT Dong N Yao YM. Novel Role of P53 in Septic Immunosuppression: Involvement in Loss and Dysfunction of CD4+ T Lymphocytes. Cell Physiol Biochem Int J Exp Cell Physiol Biochem Pharmacol (2018) 51:452–69. 10.1159/000495241 30453300

[37] Ryu HW Shin DH Lee DH Choi J Han G Lee KY. HDAC6 Deacetylates P53 at Lysines 381/382 and Differentially Coordinates P53-Induced Apoptosis. Cancer Lett (2017) 391:162–71. 10.1016/j.canlet.2017.01.033 28153791

[38] Liu Y Tavana O Gu W. P53 Modifications: Exquisite Decorations of the Powerful Guardian. J Mol Cell Biol (2019) 11:564–77. 10.1093/jmcb/mjz060 PMC6736412 31282934

[39] Yoshida M Furumai R Nishiyama M Komatsu Y Nishino N Horinouchi S. Histone Deacetylase as a New Target for Cancer Chemotherapy. Cancer Chemother Pharmacol (2001) 48 Suppl 1:S20–6. 10.1007/s002800100300 11587361

[40] van Leeuwen I Lain S. Sirtuins and P53. Adv Cancer Res (2009) 102:171–95. 10.1016/S0065-230X(09)02005-3 19595309

[41] Xu SQ lI LL Wu J An S Fang HH Han YY. Melatonin Attenuates Sepsis-Induced Small-Intestine Injury by Upregulating SIRT3-Mediated Oxidative-Stress Inhibition, Mitochondrial Protection, and Autophagy Induction. Front Immunol (2021) 12:625627. 10.3389/fimmu.2021.625627 33790896 PMC8006917

[42] Li H Peng X Wang Y Cao S Xiong L Fan J. Atg5-Mediated Autophagy Deficiency in Proximal Tubules Promotes Cell Cycle G2/M Arrest and Renal Fibrosis. Autophagy (2016) 12:1472–86. 10.1080/15548627.2016.1190071 PMC5082781 27304991

[43] Ishihara M Urushido M Hamada K Matsumoto T Shimamura Y Ogata K. Sestrin-2 and BNIP3 Regulate Autophagy and Mitophagy in Renal Tubular Cells in Acute Kidney Injury. Am J Physiol Renal Physiol (2013) 305:F495–509. 10.1152/ajprenal.00642.2012 23698117

[44] Tasdemir E Maiuri MC Galluzzi L Vitale I Djavaheri-Mergny M D’Amelio M. Regulation of Autophagy by Cytoplasmic P53. Nat Cell Biol (2008) 10:676–87. 10.1038/ncb1730 PMC2676564 18454141

[45] Hoshino A Ariyoshi M Okawa Y Kaimoto S Uchihashi M Fukai K. Inhibition of P53 Preserves Parkin-Mediated Mitophagy and Pancreatic β-Cell Function in Diabetes. Proc Natl Acad Sci USA (2014) 111:3116–21. 10.1073/pnas.1318951111 PMC3939874 24516131

[46] Song YM Lee WK Lee YH Kang ES Cha BS Lee BW. Metformin Restores Parkin-Mediated Mitophagy, Suppressed by Cytosolic P53. Int J Mol Sci (2016) 17(1):122. 10.3390/ijms17010122 PMC4730363 26784190

[47] Hoshino A Mita Y Okawa Y Ariyoshi M Iwai-Kanai E Ueyama T. Cytosolic P53 Inhibits Parkin-Mediated Mitophagy and Promotes Mitochondrial Dysfunction in the Mouse Heart. Nat Commun (2013) 4:2308. 10.1038/ncomms3308 23917356

[48] Tripathi R Ash D Shaha C. Beclin-1-P53 Interaction Is Crucial for Cell Fate Determination in Embryonal Carcinoma Cells. J Cell And Mol Med (2014) 18:2275–86. 10.1111/jcmm.12386 PMC4224560 25208472

[49] Holthoff JH Wang Z Seely KA Gokden N Mayeux PR. Resveratrol Improves Renal Microcirculation, Protects the Tubular Epithelium, and Prolongs Survival in a Mouse Model of Sepsis-Induced Acute Kidney Injury. Kidney Int (2012) 81:370–8. 10.1038/ki.2011.347 PMC3326404 21975863

[50] Zhang ZS Zhao HL Yang GM Zang JT Zheng DY Duan CY. Role of Resveratrol in Protecting Vasodilatation Function in Septic Shock Rats and its Mechanism. J Trauma Acute Care Surg (2019) 87:1336–45. 10.1097/TA.0000000000002466 31389921

