# Supplementary material for: p53 Deacetylation Alleviates Sepsis-Induced Acute Kidney Injury by Promoting Autophagy
Source: Front Immunol. 2021 Jul 14;12:685523. doi: 10.3389/fimmu.2021.685523 (PMC8318785; doi:10.3389/fimmu.2021.685523)
Supplement: Supplementary file 1 [file Image_1.pdf]

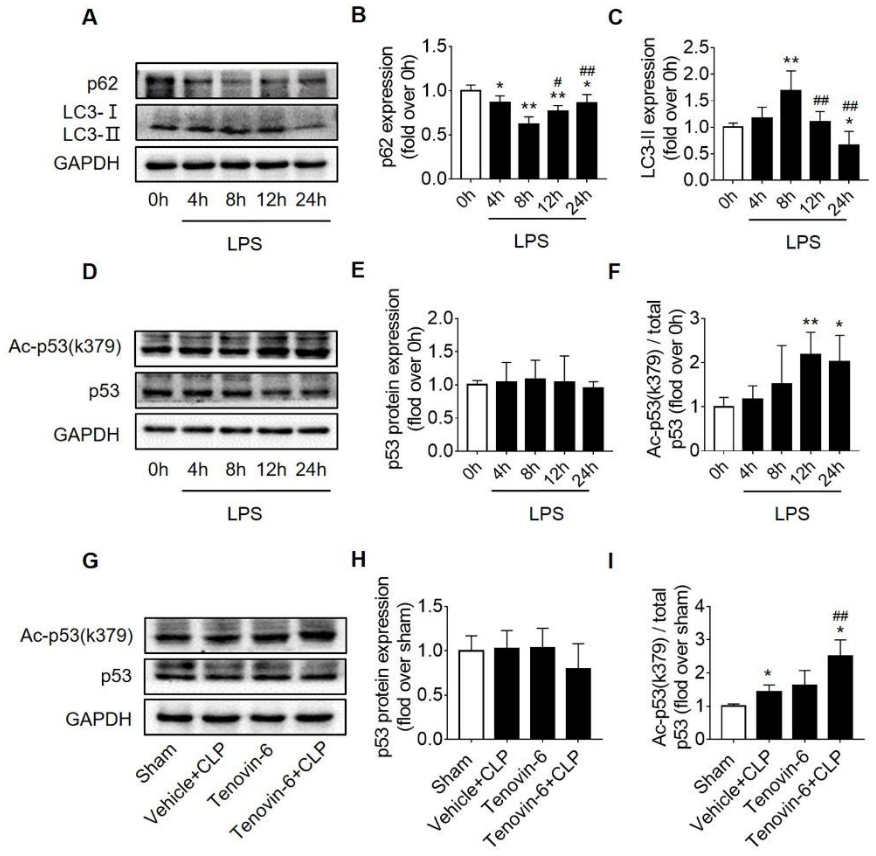

SFigure 1. Determination of autophagy and p53 acetylation in LPS-induced sepsis (A-F), and the effect of Tenovin-6 on p53 acetylation in CLP-induced sepsis (G-I). (A) Representative western blot showing the p62 and LC3II protein expression levels in the renal cortex following LPS-induced sepsis. GAPDH was used as an internal reference. (B and C) Densitometric analyses of p62 and LC3II protein expression. n=3-4. $^{*}p < 0.05$, $^{**}p < 0.01$ *vs.* 0 h group; $^{\#}p < 0.05$, $^{\#\#}p < 0.01$ *vs*. 8 h group. (D) Levels of total protein expression and acetylated p53 (ac-p53) at lysine site K379 in LPS-induced sepsis. (E and F) Densitometric analyses of p53 protein expression and acetylation at lysine site K379 in LPS-induced sepsis. n=3-4. $^{*}p < 0.05$, $^{**}p < 0.01$ *vs.* 0 h group. (G) Levels of total protein expression and acetylated p53 (ac-p53) at lysine site K379 in CLP-induced sepsis. (H and I) Densitometric analyses of p53 protein expression and acetylation at lysine site K379 in CLP-induced sepsis. n=5-6. $^{*}p < 0.05$ versus sham group; $^{\#\#}p < 0.01$ *vs*. Vehicle+CLP group. LPS: lipopolysaccharide; LC3II: Microtubule-associated protein 1A/1B-light chain 3; GAPDH, glyceraldehyde 3-phosphate dehydrogenase; CLP: cecal ligation and puncture.
