# Supplementary material for: p53 Deacetylation Alleviates Sepsis-Induced Acute Kidney Injury by Promoting Autophagy
Source: Front Immunol. 2021 Jul 14;12:685523. doi: 10.3389/fimmu.2021.685523 (PMC8318785; doi:10.3389/fimmu.2021.685523)
Supplement: Supplementary file 2 [file Image_2.pdf]

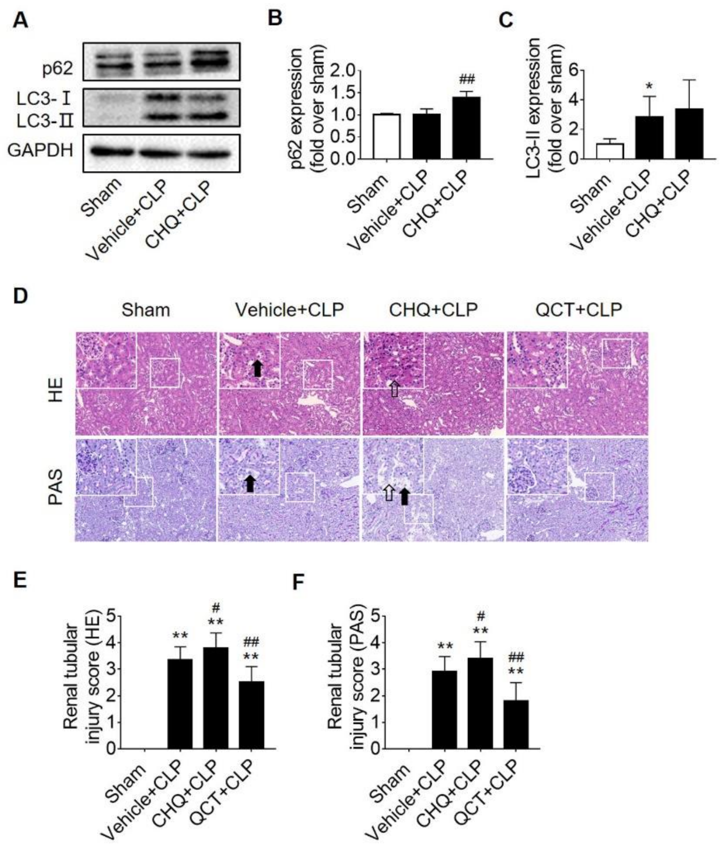

SFigure 2. Effects of autophagy inhibitor CHQ and Sirt1 activator QCT on kidney injury in CLP-induced sepsis. (A) Representative western blot showing the p62 and LC3II protein expression levels in the renal cortex following CLP-induced sepsis. GAPDH was used as an internal reference. (B and C) Densitometric analyses of p62 and LC3II protein expression. n=3-6. (D) Hematoxylin-eosin (HE) staining (upper panel: 200×; inset: 400×) and periodic acid-Schiff (PAS) staining of the renal cortex following CLP-induced sepsis. Black thick arrows: Nuclei of RTECs shed to lumen; White thick arrows: severe tubular damage. (E and F) The tubular damage score was evaluated based on pathological observations from HE and PAS staining. $^{*}p < 0.05$, $^{**}p < 0.01$ *vs*. sham group; $^{\#}p < 0.05$, $^{\#\#}p < 0.01$ *vs*. Vehicle+CLP group. n=5. CLP: cecal ligation and puncture; LC3II: Microtubule-associated protein 1A/1B-light chain 3; GAPDH: glyceraldehyde 3-phosphate dehydrogenase; RTEC: renal tubule epithelial cell.
