# Supplementary material for: p53 Deacetylation Alleviates Sepsis-Induced Acute Kidney Injury by Promoting Autophagy
Source: Front Immunol. 2021 Jul 14;12:685523. doi: 10.3389/fimmu.2021.685523 (PMC8318785; doi:10.3389/fimmu.2021.685523)
Supplement: Supplementary file 3 [file Image_3.pdf]

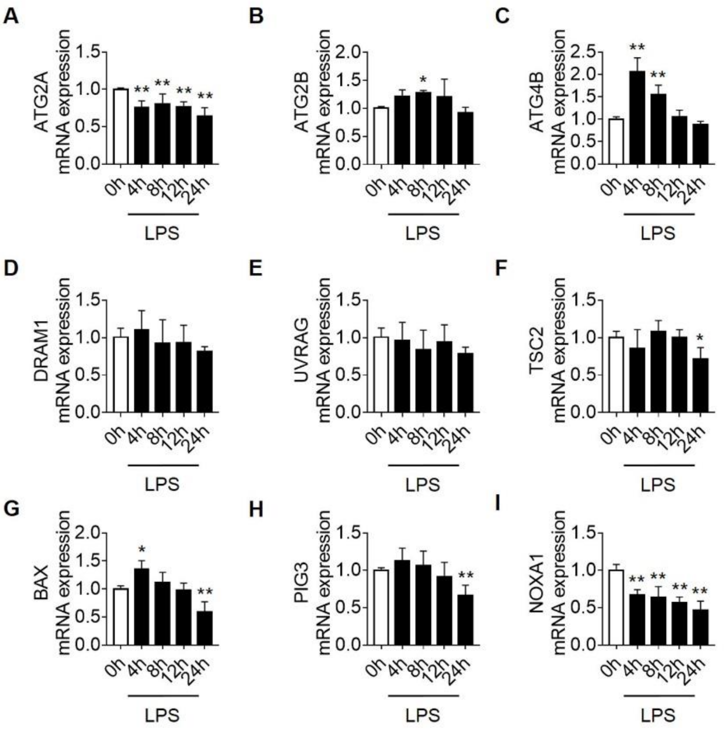

SFigure 3. Transcription levels of p53 target genes in LPS-treated HK-2 cells. (A–F) mRNA levels of autophagy-related genes ATG2A, ATG2B, ATG4B, DRAM1, UVRAG, TSC2. (G–I) mRNA levels of apoptosis-related genes BAX, PIG3 and NOXA1. n = 6. $^{*}p < 0.05$, $^{**}p < 0.01$ *vs*. 0 h group.
