# Supplementary material for: p53 Deacetylation Alleviates Sepsis-Induced Acute Kidney Injury by Promoting Autophagy
Source: Front Immunol. 2021 Jul 14;12:685523. doi: 10.3389/fimmu.2021.685523 (PMC8318785; doi:10.3389/fimmu.2021.685523)
Supplement: Supplementary file 4 [file Image_4.pdf]

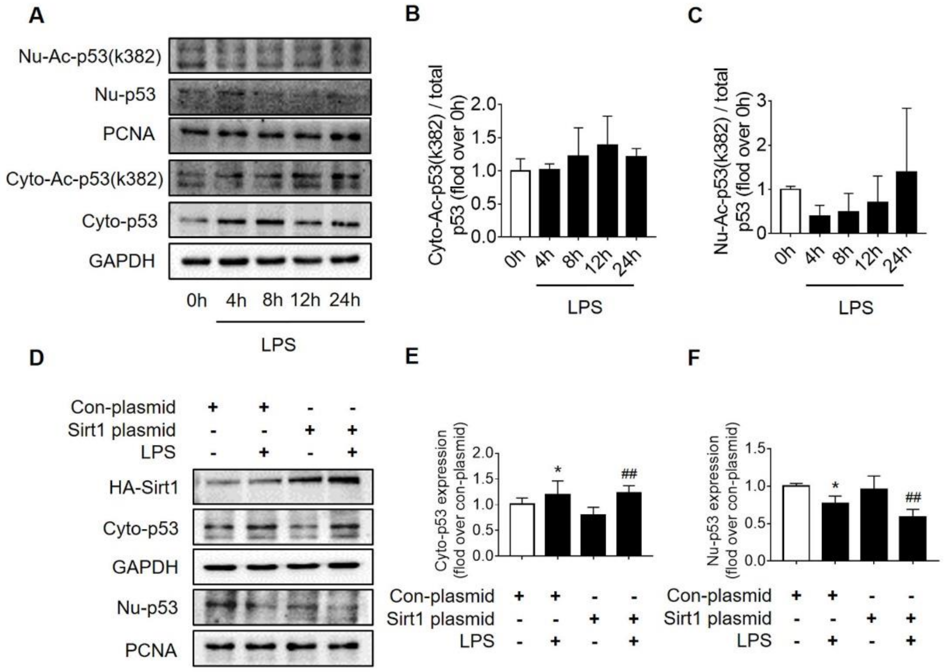

SFigure 4. The determination of cytoplasmic p53 and nuclear p53 acetylation (A-C), the effect of Sirt1 on p53 distribution (D-F). (A) Representative western blot showing the levels of p53 protein expression and acetylation at lysine site K382 in HK-2 cells following LPS treatment. GAPDH was used as an internal control of p53 total protein and cytoplasmic protein levels, and PCNA was used as an internal control for nuclear proteins. (B and C) Densitometric analyses of the levels of p53 acetylation at lysine site K382 in HK-2 cells following LPS treatment. (D) Representative western blot of HA-Sirt1, cytoplasmic p53 and nuclear p53 in LPS-treated HK-2 cells. GAPDH was used as an internal control of cytoplasmic protein levels, and PCNA was used as an internal control for nuclear proteins. (E and F) Densitometric analyses of cytoplasmic protein level (cyto-p53) and nuclear protein level (Nu-p53). n = 3-4. $^{*}p < 0.05$ *vs*. Con-plasmid group; $^{\#\#}p < 0.01$ *vs*. Con-plasmid+LPS group. LPS: lipopolysaccharide; PCNA: proliferating cell nuclear antigen; GAPDH, glyceraldehyde 3-phosphate dehydrogenase; CLP: cecal ligation and puncture.
