# Supplementary material for: p53 Deacetylation Alleviates Sepsis-Induced Acute Kidney Injury by Promoting Autophagy
Source: Front Immunol. 2021 Jul 14;12:685523. doi: 10.3389/fimmu.2021.685523 (PMC8318785; doi:10.3389/fimmu.2021.685523)
Supplement: Supplementary file 5 [file Image_5.pdf]

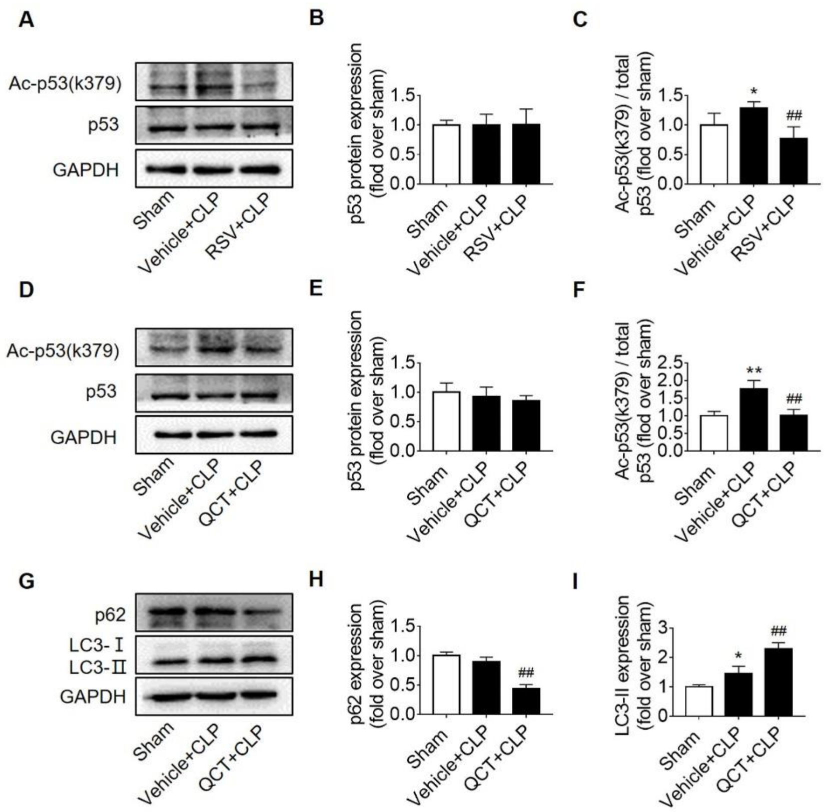

SFigure 5. Effects of Sirt1 activators RSV and QCT on p53 acetylation and autophagy in renal tissure from CLP-induced sepsis. (A) Levels of total protein expression and acetylated p53 (ac-p53) at lysine site K379 in CLP-induced sepsis. (B and C) Densitometric analyses of the levels of p53 protein expression and acetylation at lysine site K379 in CLP-induced sepsis. (D) Levels of total protein expression and acetylated p53 (ac-p53) at lysine site K379 in CLP-induced sepsis. (E and F) Densitometric analyses of the levels of p53 protein expression and acetylation at lysine site K379 in CLP-induced sepsis. (G) Representative western blot showing the p62 and LC3II protein expression levels in the renal cortex following CLP-induced sepsis. GAPDH was used as an internal reference. (H and I) Densitometric analyses of p62 and LC3II protein expression. n=3-4. $^{*}p < 0.05$, $^{**}p < 0.01$ *vs*. sham group; $^{\#\#}p < 0.01$ *vs*. Vehicle+CLP group. CLP: cecal ligation and puncture; LC3II: Microtubule-associated protein 1A/1B-light chain 3; GAPDH: glyceraldehyde 3-phosphate dehydrogenase.
